# Additional Effects of 1‐Hz rTMS Combined With Tai Chi Chuan on Dual‐Task Performance Among Older Adults With Sleep Disorders and MCI: An fNIRS‐Based Randomized Controlled Trial

**DOI:** 10.1002/brb3.71743

**Published:** 2026-09-14

**Authors:** Zhenxing Guo, Kaifeng Xu, Linxin Bai, Yuxuan Sun, Lin Zhang, Jiahui Gao, Lidian Chen, Zhizhen Liu

**Affiliations:** ^1^ College of Rehabilitation Medicine Fujian University of Traditional Chinese Medicine Fuzhou Fujian China; ^2^ College of Pharmacy Fujian University of Traditional Chinese Medicine Fuzhou Fujian China; ^3^ National‐Local Joint Engineering Research Center of Rehabilitation Medicine Technology Fujian University of Traditional Chinese Medicine Fuzhou Fujian China; ^4^ Key Laboratory of Cognitive Rehabilitation of Fujian Province Fuzhou Fujian China

**Keywords:** dual‐task walking, functional near‐infrared spectroscopy, repetitive transcranial magnetic stimulation, Tai Chi Chuan

## Abstract

**Objective:**

This study aimed to determine whether 1‐Hz repetitive transcranial magnetic stimulation (rTMS) combined with Tai Chi Chuan exerts an additional effect on dual‐task performance in older adults with sleep disorders and mild cognitive impairment (MCI) and to explore the underlying cerebral hemodynamic mechanisms.

**Methods:**

A total of 110 older adults with sleep disorders and MCI were randomly assigned to receive 6 weeks of either 1‐Hz rTMS or sham stimulation in combination with Tai Chi Chuan. Dual‐task performance, gait and balance outcomes, and task‐evoked cerebral hemodynamic responses and functional connectivity measured using functional near‐infrared spectroscopy (fNIRS) were assessed at baseline and after the intervention.

**Results:**

Compared with sham, active rTMS combined with Tai Chi Chuan significantly improved dual‐task performance (*p* = 0.005). During dual‐tasking, greater activation was observed in the left dorsolateral prefrontal cortex (DLPFC) and frontal pole (both *p* = 0.003), along with stronger connectivity between the premotor area and frontal pole (*p *< 0.001) and between the premotor area and orbitofrontal cortex (OFC; *p* = 0.001). Changes in left DLPFC activation correlated with changes in subtraction accuracy (*r* = 0.313, *p* = 0.034). Furthermore, changes in right DLPFC–right OFC connectivity correlated with changes in subtraction accuracy (*r* = 0.382, *p* = 0.009).

**Conclusions:**

When added to Tai Chi Chuan, 1‐Hz active rTMS improved dual‐task performance more than sham rTMS in older adults with sleep disorders and MCI. The observed changes in prefrontal and premotor activation and functional connectivity may contribute to this additional effect.

**Trial Registration:**

Chinese Clinical Trial Registry (ChiCTR2200063274). Registered on September 2, 2022; https://www.chictr.org.cn/showproj.html?proj=176982.

## Graphical abstract

1‐Hz rTMS enhances Tai Chi–induced improvements in dual‐task performance in older adults with sleep disorders and MCI, potentially via increased activation and functional connectivity within prefrontal and premotor regions.

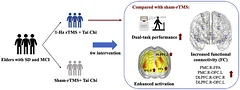

## 1 Introduction

In daily life, many activities are performed under dual‐task conditions, requiring individuals to allocate attention simultaneously to competing motor and cognitive demands (Li et al. 2023). Dual‐task performance engages higher order cognitive processes that regulate behavior and is commonly used to assess attentional allocation (Iwasaki et al. 2022). Sleep disturbances may further compromise attentional control by impairing glymphatic clearance and disrupting neuronal function and interregional communication (Ehgoetz Martens et al. 2020; Jiang‐Xie et al. 2024). Mild cognitive impairment (MCI) is similarly associated with deficits in executive function and working memory that may constrain performance under concurrent motor and cognitive demands (Kuo et al. 2022; Watanabe and Funahashi 2014). The coexistence of sleep disorders and MCI may therefore compound dual‐task impairment through their convergent effects on executive control and fronto‐motor communication (Lee et al. 2024; Palmer et al. 2018). Tai Chi Chuan is a traditional Chinese mind‐body exercise that integrates musculoskeletal, sensory, and cognitive processes. It involves controlled, self‐initiated movements coordinated with breathing, including center‐of‐gravity displacement, weight shifting, trunk rotation, and eye‐hand coordination (Chen et al. 2023). Compared with exercise programs designed primarily to improve aerobic capacity or muscle strength, Tai Chi Chuan concurrently engages balance control, motor planning, cognitive processing, and mind‐body coordination. In this study, we used the Yang‐style 24‐form Tai Chi Chuan regimen, a standardized sequence with low‐impact movements that can be adapted for older adults. Tai Chi Chuan may improve cognitive‐motor control and dual‐task performance in older adults (Ito 2024; Li et al. 2023). However, sustained practice may be limited by movement complexity, health constraints, access to classes, and insufficient social support (Du et al. 2023).

Recent studies have begun to combine Tai Chi Chuan with non‐invasive brain stimulation, including repetitive transcranial magnetic stimulation (rTMS) and transcranial direct current stimulation (He et al. 2024; Liao et al. 2021). Low‐frequency rTMS applied to the dorsolateral prefrontal cortex (DLPFC) may modulate neural activity and plasticity within distributed cortical networks (Zhang et al. 2022), although the direction of its local and network‐level effects is context dependent. Such neuromodulation may complement the cognitive‐motor training provided by Tai Chi Chuan. In our previous randomized trial, adding 1‐Hz rTMS to a common Tai Chi Chuan program resulted in greater improvements in sleep quality and cognitive function than sham rTMS plus the same Tai Chi Chuan program (Liu et al. 2025). However, the effects of rTMS combined with Tai Chi Chuan on dual‐task performance in older adults with sleep disorders and MCI, as well as the underlying neuroimaging mechanisms, have yet to be fully elucidated.

The prefrontal cortex supports attentional allocation and cognitive control during dual‐task performance (Watanabe and Funahashi 2014). Dual‐task walking additionally requires coordination between prefrontal and motor regions (Ding et al. 2024). Functional near‐infrared spectroscopy (fNIRS) enables non‐invasive measurement of cortical hemodynamic responses during walking and has been used to examine prefrontal activity during cognitive‐motor dual tasks (Chen et al. 2020). Research has indicated that cognitive task performance during dual‐task walking in older adults is related to activation levels in the prefrontal cortex and sensorimotor areas (Baek et al. 2023; Ding et al. 2024). In healthy adults, greater dual‐task costs have also been associated with prefrontal overactivation and reduced network efficiency across prefrontal, motor and sensorimotor regions (Ding et al. 2024). Thus, changes in the cerebral hemodynamics in the prefrontal and motor areas may be key factors in improving dual‐task performance in older adults with sleep disorders and MCI.

Therefore, we hypothesized that 1‐Hz rTMS combined with Tai Chi Chuan would exert an additional effect on dual‐task performance in older adults with sleep disorders and MCI, with changes in dual‐task performance linked to alterations in the activation and functional connectivity within the prefrontal and motor areas. To validate this hypothesis, we conducted a 6‐week, double‐blind, sham‐controlled randomized trial to determine whether 1‐Hz rTMS targeting the right DLPFC provides greater improvements in dual‐task performance than sham rTMS does. Furthermore, we utilized fNIRS to explore the cerebral hemodynamic characteristics underlying the effects of combining rTMS with Tai Chi Chuan during dual‐tasking. This design allowed us to estimate the additional effect of active rTMS and examine its cortical correlates during dual‐task walking.

## 2 Methods

### 2.1 Study Design and Participants

**FIGURE 1:**
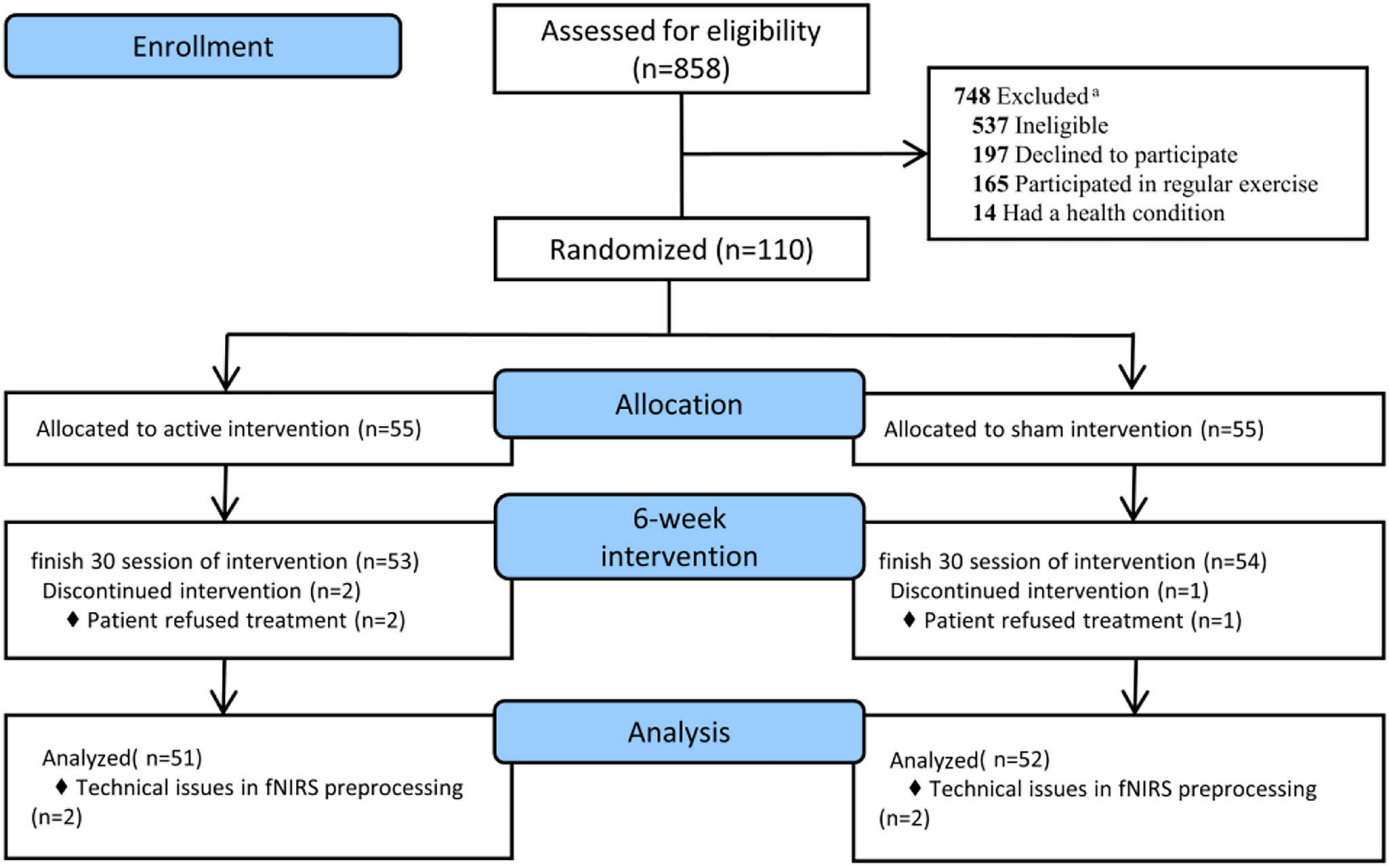
**Trial flow chart**. fNIRS, functional near‐infrared spectroscopy; rTMS, repetitive transcranial magnetic stimulation. ^a^Participants could be excluded for more than 1 reason.

We conducted a two‐arm, sham‐controlled, assessor‐masked randomized clinical trial that was approved by the ethics committee of the Affiliated Rehabilitation Hospital of Fujian University of Traditional Chinese Medicine (approval number: 2022KY‐024‐01). All participants provided written informed consent. Eligible individuals were randomly assigned to two groups: the experimental group (Tai Chi Chuan + 1‐Hz rTMS) and the sham group (Tai Chi Chuan + sham rTMS). The flowchart of this trial is presented in Figure 1. The participants completed functional assessments at baseline and after the 6‐week intervention, which included evaluations of dual‐task performance, gait balance, and changes in cerebral hemodynamics during dual‐task execution. The study is registered with the Chinese Clinical Trial Registry (No. ChiCTR2200063274).

The participants were recruited from a preliminary project conducted at a university hospital in China between October 2022 and February 2024. The total sample size was 110 participants (Project No. 2022J06028). Among these individuals, 103 participants successfully completed the dual‐task assessment and had fNIRS data. The effect size was determined on the basis of a previous trial (Schneider et al. 2021). The primary endpoint of dual‐task performance in the present analysis was the change in serial‐7 subtraction accuracy during dual‐task walking, calculated as the post‐intervention value minus the baseline value. With a significance level set at *α* = 0.05, the statistical power of this study was calculated to be 0.94 via G Power 3.1 software. Therefore, this study possesses high statistical power.

A total of 110 participants were recruited from a community in Fuzhou, China. The inclusion criteria were as follows: aged 60–75 years; diagnosed with sleep disorders; and MCI without dementia. Individuals who had engaged in regular moderate‐intensity exercise or Tai Chi (more than three times per week for more than 20 min per session) in the past 3 months were not eligible for inclusion. After a thorough explanation of the study procedures, all participants provided written informed consent.

Sleep disorder was diagnosed according to the International Classification of Sleep Disorders, Third Edition (ICSD‐3). Eligible participants had one or more sleep‐related complaints despite adequate sleep opportunity, including difficulty initiating sleep, difficulty maintaining sleep, or early‐morning awakening. These symptoms were accompanied by daytime impairment, occurred at least three times per week for at least 3 months, and were accompanied by a Pittsburgh Sleep Quality Index (PSQI) score of 6 or higher. MCI was diagnosed according to Petersen's criteria, including self‐, informant‐, or clinician‐reported memory decline, a Montreal Cognitive Assessment‐Fuzhou version (MoCA‐Fuzhou) score greater than 18 and not higher than 25, preserved independence in daily living, and absence of dementia. All enrolled participants met Petersen's criteria for MCI without dementia; no cognitively normal participants were enrolled.

The exclusion criteria were as follows: (1) a Geriatric Depression Scale‐15 (GDS‐15) score of ≥9 or use of any antipsychotic medication in the past month; (2) major confounding conditions causing sleep disorders, such as severe chronic illnesses or pain disorders; (3) cognitive impairment due to other causes; (4) any physical disabilities that hinder participation in Tai Chi or rTMS; and (5) regular noninvasive stimulation or hypnotic medication treatment in the 2 weeks prior to enrollment.

### 2.2 Randomization and Blinding

The study participants were randomly assigned to the experimental group (Tai Chi Chuan + 1‐Hz rTMS) or the control group (Tai Chi Chuan + sham rTMS) at a 1:1 ratio. The allocation sequence was computer‐generated using blocks of four by an independent staff member who did not participate in the participant recruitment, interventions, outcome assessments, or statistical analysis. Participants, outcome assessors, and statistical analysts were blinded to group allocation; intervention therapists were not blinded because they operated the active or sham coil.

### 2.3 Procedures

#### 2.3.1 Tai Chi Chuan

Tai Chi Chuan training was conducted every morning for 6 weeks, with five 60‐min sessions per week. We used the Yang‐style 24‐form Tai Chi Chuan regimen, which is widely practiced and commonly used in research. Training sessions were led by certified instructors with at least 5 years of teaching experience. During each session, instructors provided on‐site guidance, corrected improper movements in real time, and emphasized both movement accuracy and mind‐body engagement. Researchers attended and supervised all training sessions. Attendance was recorded using an on‐site sign‐in system. Participants who could not attend a scheduled group session were required to request leave in advance and provide a reason. They were also instructed to practice at home using the teaching video for that day and to submit recorded practice videos to the Tai Chi Chuan coach for feedback. Training was conducted at nearby and suitable venues to improve participation and adherence.

#### 2.3.2 rTMS

To enable individualized rTMS targeting, all participants underwent baseline T1‐weighted structural MRI using a 3.0‐T Siemens Prisma scanner (Siemens, Erlangen, Germany) with an eight‐channel head coil. The T1‐weighted sequence used a repetition time of 2300 ms, an echo time of 2.48 ms, and a flip angle of 8°. The field of view was 230 × 230 mm^2^, with a matrix size of 256 × 256, a voxel size of 0.9 × 0.9 × 1.0 mm^3^, a slice thickness of 1.0 mm, and 176 slices. Individual T1‐weighted images were imported into the Brainsight version 2 neuronavigation system (Rogue Research, Montreal, Canada). Head‐to‐MRI registration was performed using the nasion and bilateral tragus points. The individualized stimulation target was defined within the right middle frontal gyrus, corresponding to the right DLPFC, according to anatomical landmarks on each participant's structural image. rTMS was delivered using a Rapid^2^ stimulator (Magstim, UK) equipped with a standard 70‐mm figure‐of‐eight coil. Neuronavigation guided coil placement over the individualized right DLPFC target. Once the target position and coil orientation were achieved, the coil was fixed in place for stimulation. The resting motor threshold was determined from motor responses of the abductor pollicis brevis muscle, and the stimulation intensity was set at 80% of the resting motor threshold. Participants received either active or sham stimulation for 20 min per session, five sessions per week for 6 weeks, with sessions scheduled in the evening. In the active‐rTMS group, stimulation was delivered continuously at 1 Hz, with a 1‐s interstimulus interval, for a total of 1200 pulses per session. The sham‐rTMS group received stimulation according to the same schedule using a sham coil with comparable physical characteristics. All rTMS sessions were administered by qualified rehabilitation therapists.

### 2.4 Outcome Measures

#### 2.4.1 Dual‐Task Performance

**FIGURE 2:**
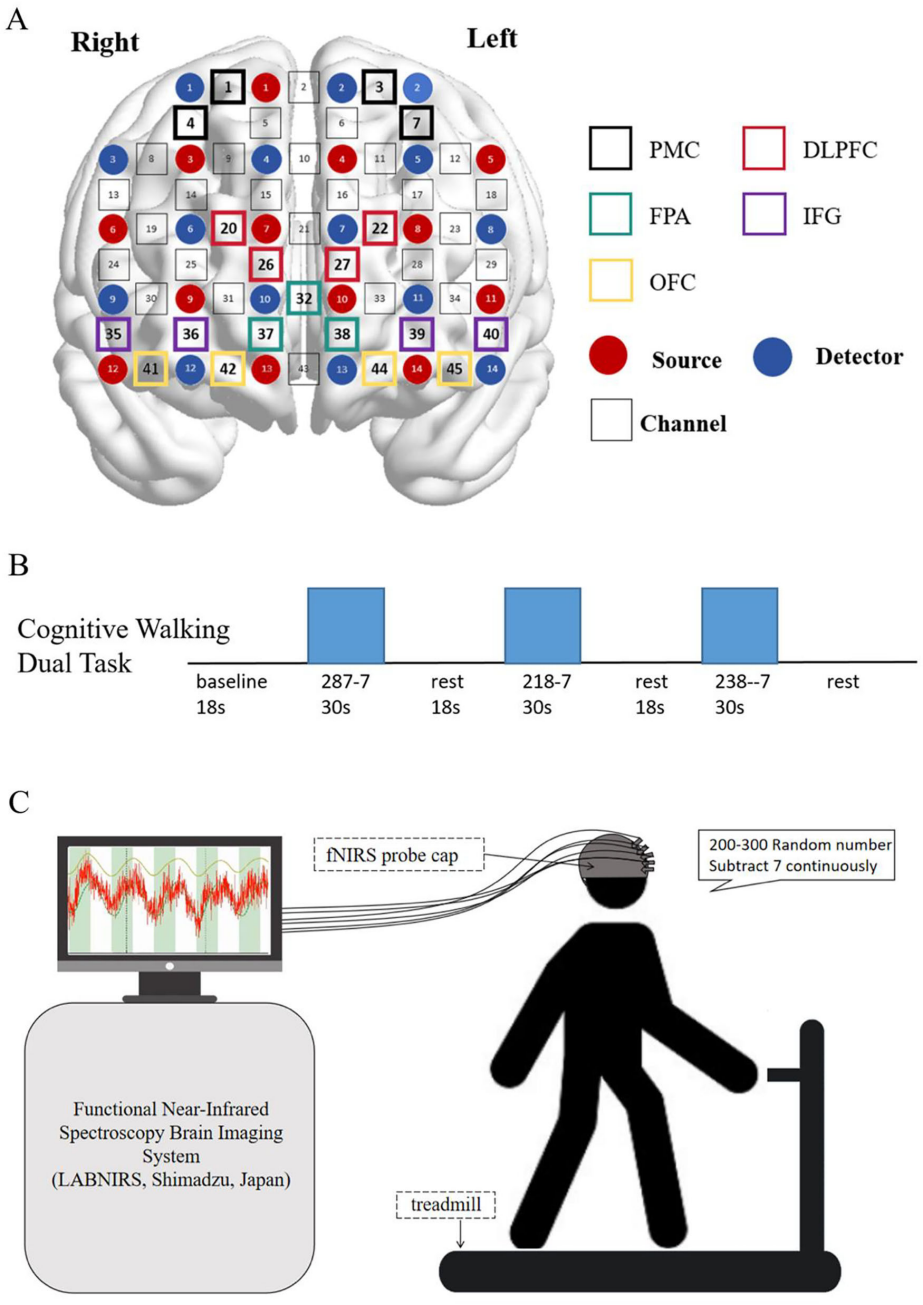
**fNIRS measurement setup and dual‐task paradigm**. (A) The distribution map of functional near‐infrared imaging channels. (B) Diagram illustrating the dual‐task subtracting 7 experimental procedure. (C) Schematic diagram of the experiment showing the execution of the dual‐task subtraction of 7 during treadmill walking while monitoring cerebral hemodynamics with functional near‐infrared spectroscopy (fNIRS). DLPFC, dorsolateral prefrontal cortex; FPA, frontal pole area; IFG, inferior frontal gyrus; OFC, orbitofrontal cortex; PMC, premotor cortex.

The participants were instructed to walk on a treadmill at their self‐selected comfortable pace while simultaneously completing a cognitive task that involved subtracting 7 from a series of randomly generated numbers ranging from 200 to 300. At each assessment, participants first walked on the treadmill at the lowest speed, which was gradually increased until they identified a comfortable pace comparable to their usual walking speed. The individually selected speed was recorded and maintained throughout all subsequent dual‐task trials within that assessment. Participants were instructed to walk at this individualized pace while performing serial‐7 subtraction, with neither the walking nor the cognitive task given priority. Before formal testing, participants completed practice trials to familiarize themselves with the treadmill‐walking and serial‐subtraction procedures and to ensure that they understood the standardized task instructions. Each session consisted of three blocks, with each block lasting 30 s and separated by 18 s of rest in addition to 18 s of rest at the beginning and end. The experimenter recorded participants' performance on the cognitive task throughout the dual‐task execution, noting the total number of responses and the number of correct responses for the subtraction task (Figure 2). Prior to the formal testing, the participants practiced the task until they fully understood the requirements. They were instructed to adhere strictly to the experimenter's directions during the tasks. The formula for calculating cognitive dual‐task performance was as follows: accuracy rate in subtracting 7 = (number of accurate responses/numbers of total responses) × 100%. The pre‐specified primary endpoint for dual‐task performance was the change in this accuracy rate from baseline to 6 weeks. During walking, a lower accuracy rate in subtracting 7 indicated a reduced ability to allocate attention (Baek et al. 2023).

#### 2.4.2 Brief Balance Evaluation Systems Test (Brief‐BESTest)

This test was utilized to assess changes in gait balance ability (Baranes et al. 2024). The Brief‐BESTest, which was developed by Jacobs JV and his team at the University of Vermont on the basis of the original BESTest, assesses six aspects: stability of the trunk/hip, forward reach with both arms, single‐leg stance (left and right legs), number of compensatory steps (left and right sides), standing on a foam board with eyes closed, and the timed get‐up‐and‐go test. Scores on the Brief‐BESTest range from 0 to 24, with lower scores indicating poorer gait balance ability.

#### 2.4.3 Sleep Quality

Sleep quality was assessed using the PSQI, which evaluates seven domains of sleep over the past month. The total score ranges from 0 to 21, with higher scores indicating poorer sleep quality.

### 2.5 fNIRS Data Acquisition and Processing

#### 2.5.1 fNIRS Measurement

Hemodynamic signals were acquired simultaneously from each dyad during the cognitive walking dual task via a multichannel high‐speed continuous wave system (LABNIRS, Shimadzu Corp, Kyoto, Japan), which consists of different laser diodes with wavelengths of 780, 805, and 830 nm. A total of 45 channels (CHs) were created via 14 sources and 14 detectors, with a sampling rate of 22.22 Hz. Using a three‐dimensional digitizer, we obtained the coordinates for all probe and anatomical landmark locations (Cz, Nz, AL, and AR) according to the international 10–20 system. MNI coordinates for each fNIRS channel were then calculated from the source and detector positions using NIRS‐SPM software. This allowed us to determine the correspondence between channels and Brodmann areas. On the basis of their coordinate locations, channels were subsequently grouped into five anatomical regions: the premotor cortex (PMC; CH1, CH3, CH4, CH7), DLPFC (CH20, CH22, CH26, CH27), frontal pole area (FPA; CH32, CH37, CH38), inferior frontal gyrus (IFG; CH35, CH36, CH39, CH40), and orbitofrontal cortex (OFC; CH41, CH42, CH44, CH45). These regions could be further divided into left (L) and right (R) brain areas, except for the FPA (Figure 2). Detailed channel coordinates and the corresponding brain regions of interest can be found in Tables S1 and S2.

#### 2.5.2 fNIRS Data Analysis

In this study, the preprocessing and analysis of fNIRS data were performed via Homer2 toolbox (David Boas, Jay Dubb, Ted Huppert, Meryem Ayse Yucel, Boston, MA, USA) (Huppert et al. 2009). This process involved noise reduction, manual removal of bad segments, identification of faulty channels, motion artifact correction, and filtering. The filtered optical density data were ultimately converted into the concentrations of oxygenated hemoglobin, deoxygenated hemoglobin, and total hemoglobin.

Upon completion of data preprocessing, the hmrBlockAvg function in Homer2 was utilized to perform block averaging on the time series segments, thus enabling the calculation of individual hemoglobin concentrations. The mean changes in hemoglobin concentrations across all individuals were subsequently averaged to obtain the group‐level mean changes in hemoglobin concentrations. To assess functional connectivity between channel pairs, correlation analysis was conducted via the MATLAB function mscohere, and the correlation coefficients for each channel pair were subjected to *z*‐transformation. Intergroup comparisons were performed via independent samples *t*‐tests (Li et al. 2022).

Following fNIRS preprocessing, we used the NIRS‐KIT toolbox in MATLAB to establish a general linear model (GLM) for individual‐level statistical analysis. The model yielded activation *β* values for each experimental condition and enabled assessment of task‐related neural activity (Hou et al. 2021). To provide a clearer representation of regional brain activation, a contrast vector was established, which was calculated as the *β* value of oxygenated hemoglobin during the task state minus the *β* value during the rest state. This vector yielded *β* values that represent regional brain activation. This approach enabled the assessment of variations in brain activity under different conditions, thus providing critical quantitative data for understanding the neural mechanisms associated with the task.

### 2.6 Statistical Analysis

This study used the IBM SPSS Statistics software version 24.0 for data analysis, with the significance level set to 0.05 for all analyses. Continuous variables are reported as the means (standard deviations, SDs) or medians (interquartile ranges, IQRs). We performed between‐group comparisons of baseline characteristics and examined changes in dual‐task performance (the accuracy rate), Brief‐BESTest scores, *β* values, and the correlation coefficients for each channel pair by comparing values before and after the intervention (post‐pre). For each participant, change scores were calculated as post‐intervention values minus baseline values (*Δ* = post‐pre). The primary between‐group comparison assessed whether the mean change differed between the active‐rTMS and sham‐rTMS groups using an independent‐samples *t*‐test or Mann–Whitney *U* test, as appropriate. In this two‐group design with two measurement time points, the between‐group comparison of change scores evaluates whether changes over time differed between groups. Categorical variables are presented as counts and percentages and were analyzed by using the *χ* ^2^ test. Additionally, to control for the potential Type I error associated with multiple comparisons, we employed the Benjamini–Hochberg procedure to maintain the false discovery rate (FDR) at an alpha level of 0.05. The primary analysis used complete cases with available baseline and post‐intervention dual‐task and fNIRS data. Participants who withdrew before receiving stimulation and participants with unusable fNIRS data were excluded from the final fNIRS‐based analysis, and missing outcome data were not imputed.

Associations between changes in dual‐task performance and changes in cerebral hemodynamics were examined using partial Pearson correlations within the experimental group. The analyses used change scores for *β* values, channel‐pair correlation coefficients under dual‐task conditions, and behavioral outcomes. Covariates were the GDS‐15 score, Hamilton Depression Rating Scale (HAMD) total score, PSQI total score, history of hyperlipidemia, and MoCA total score.

## 3 Results

### 3.1 Characteristics of Participants

We screened 858 potential participants and ultimately enrolled 110 older adults with sleep disorders and MCI who met the inclusion criteria and provided informed consent. Following baseline assessments, these participants were randomly assigned to the experimental group (Tai Chi Chuan + 1‐Hz rTMS) or the control group (Tai Chi Chuan + sham rTMS), with 55 participants in each group. During the intervention, three participants withdrew before completing any stimulation visits, and four additional participants were excluded due to poor fNIRS data quality. Consequently, the final analysis included 51 participants in the experimental group and 52 participants in the control group (Figure 1). The baseline demographic and clinical characteristics of the experimental group and the control group were comparable (Table 1).

**TABLE 1:** The clinical manifestations of groups receiving different intervention schemes.

Variables	Experimental (*n* = 51)	Sham (*n* = 52)	*p*
**Age, years**	67.7 ± 4.7	68.0 ± 4.7	0.882
**Female,** *n* (%)	34 (67)	29 (56)	0.257
**Education, years**	10.9 ± 2.6	11.1 ± 2.7	0.436
**Hypertension,** *n* (%)			0.907
**No**	30 (59)	30 (58)	
**Yes**	21 (41)	22 (42)	
**Hyperlipidemia,** *n* (%)			0.123
**No**	38 (75)	45 (87)	
**Yes**	13 (25)	7 (13)	
**GDS‐15, scores**	2.5 ± 1.7	2.6 ± 2.3	0.719
**HAMD, scores**	5.6 ± 3.1	5.4 ± 2.5	0.925
**MoCA, scores**	23.3 ± 1.7	22.6 ± 2.0	0.129
**PSQI, scores**	12.8 ± 2.8	12.5 ± 2.7	0.553
**DT‐Accuracy, %**	79.6 ± 18.2	77.8 ± 19.2	0.565
**Brief‐BESTest, scores**	20.0 ± 3.2	19.0 ± 4.0	0.319

*Note*: Values are mean ± standard deviation.

Abbreviations: Brief‐BESTest, brief balance evaluation systems test; DT‐Accuracy, accuracy rate of subtracting 7 under dual‐task conditions; GDS‐15, Geriatric Depression Scale‐15; HAMD, Hamilton Depression Scale; MoCA, Montreal Cognitive Assessment; PSQI, Pittsburgh Sleep Quality Index.

### 3.2 Dual‐Task Performance (Serial Subtraction of Sevens)

**FIGURE 3:**
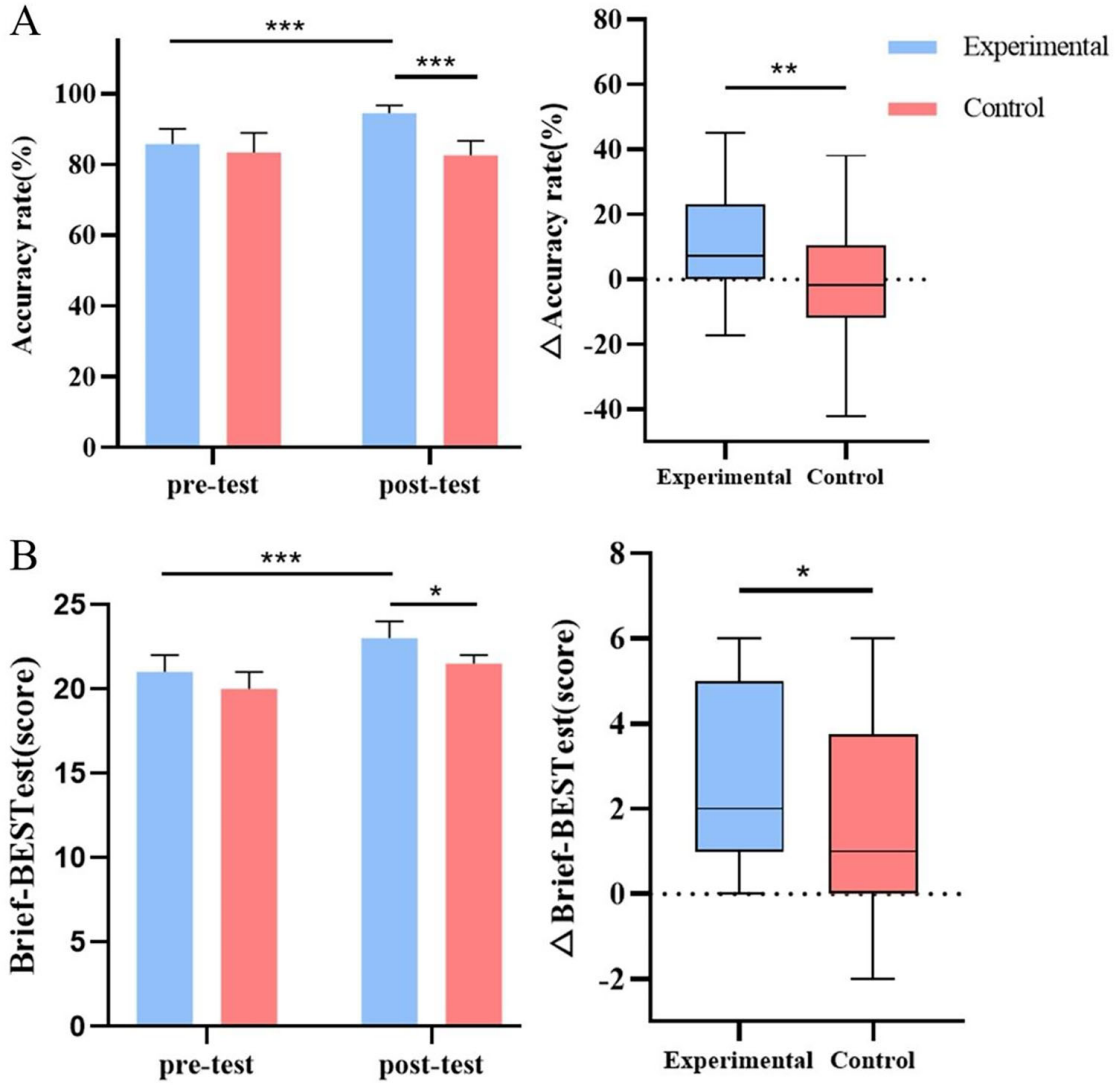
**The effects of combined rTMS and Tai Chi on dual‐task performance and gait balance**. (A) Changes in dual‐task accuracy for the reduced seven tasks before and after the intervention in both groups, as well as the intergroup difference in accuracy change (*Δ* accuracy). (B) Differences in the Brief‐BESTest scores before and after the intervention in both groups, along with the intergroup differences in the changes in the Brief‐BESTest scores (*Δ* Brief‐BESTest). *p < 0.05, **p < 0.01, ***p < 0.001; R, right; L, left; pre‐test, preintervention testing; post‐test, post‐intervention testing; *Δ*, post‐intervention minus preintervention. Brief‐BESTest, brief balance evaluation systems test.

The primary endpoint, the change in accuracy rate during the serial‐7 subtraction dual‐task walking condition, was significantly greater in the experimental group than in the control group after 6 weeks of intervention (*p* = 0.005; Figure 3A). Post‐intervention accuracy rate was also higher in the experimental group than in the control group (*p* < 0.001; Figure 3A). In addition, the experimental group showed a higher post‐intervention total response count (*p* = 0.023) and the number of correct responses (*p* = 0.001), and a greater increase in the number of correct responses (*p* = 0.038) than the control group. There was no significant between‐group difference in the change in total response count (*p* > 0.05) (Table S3).

### 3.3 Gait Balance Performance

In the experimental group, Brief‐BESTest scores increased significantly from baseline to the end of the 6‐week intervention (*p* < 0.001). Furthermore, the improvement in the Brief‐BESTest score in the experimental group was significantly greater than that in the control group (*p* = 0.021) (Figure 3B).

### 3.4 Sleep Quality

**FIGURE 4:**
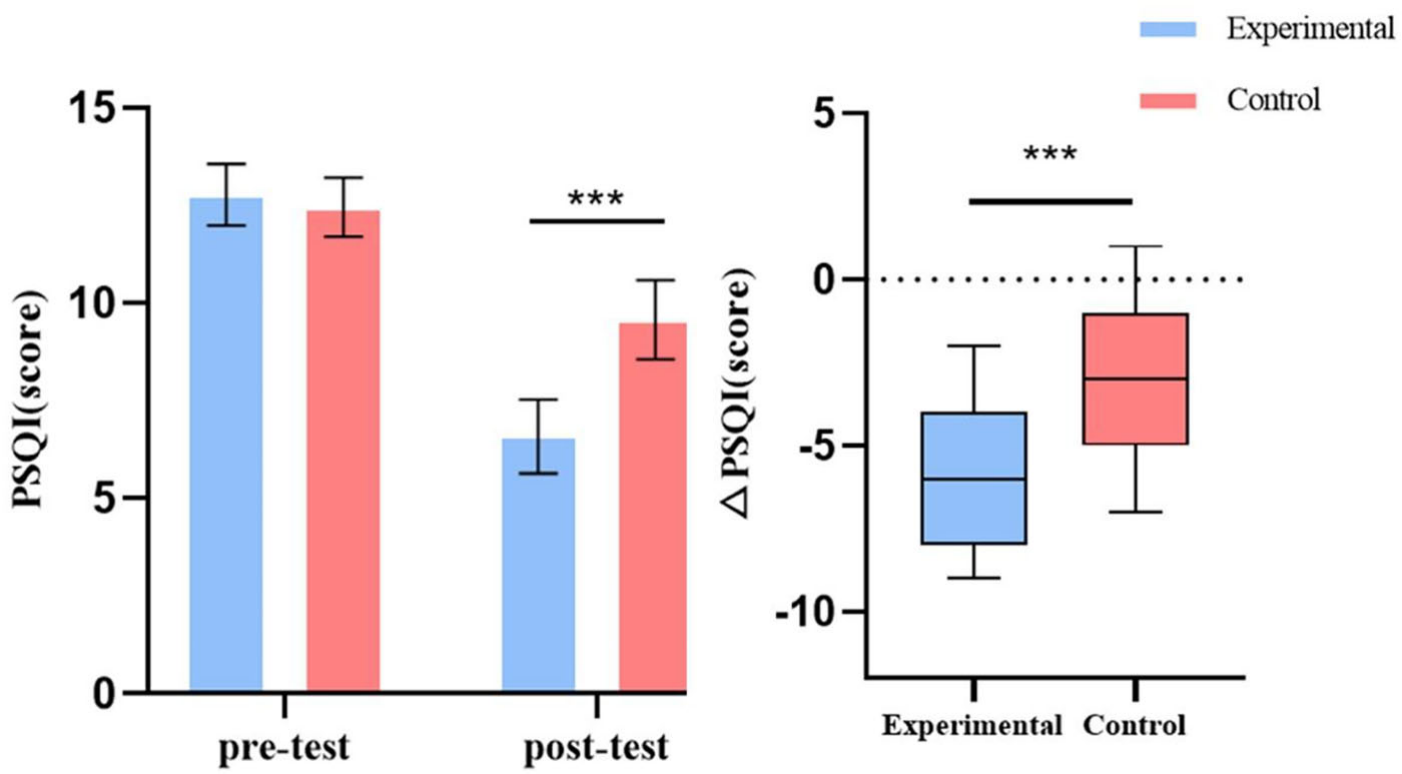
**The effects of combined rTMS and Tai Chi on Sleep Quality**. Differences in the PSQI scores before and after the intervention in both groups, along with the intergroup differences in the changes in the PSQI scores (*Δ* PSQI). PSQI, Pittsburgh Sleep Quality Index; *p < 0.05, **p < 0.01, ***p < 0.001; pre‐test, preintervention testing; post‐test, post‐intervention testing; *Δ*, post‐intervention minus preintervention.

After 6 weeks of intervention, the experimental group had significantly lower PSQI scores compared to the control group (*p* < 0.001). Furthermore, the degree of reduction in PSQI scores for the experimental group was even more pronounced, indicating that the improvement in sleep quality in the experimental group after the combined intervention was significantly greater than that in the control group (*p* < 0.001). This is consistent with the findings of our research team's previous studies (Liu et al. 2025) (Figure 4).

### 3.5 *β* Values

**FIGURE 5:**
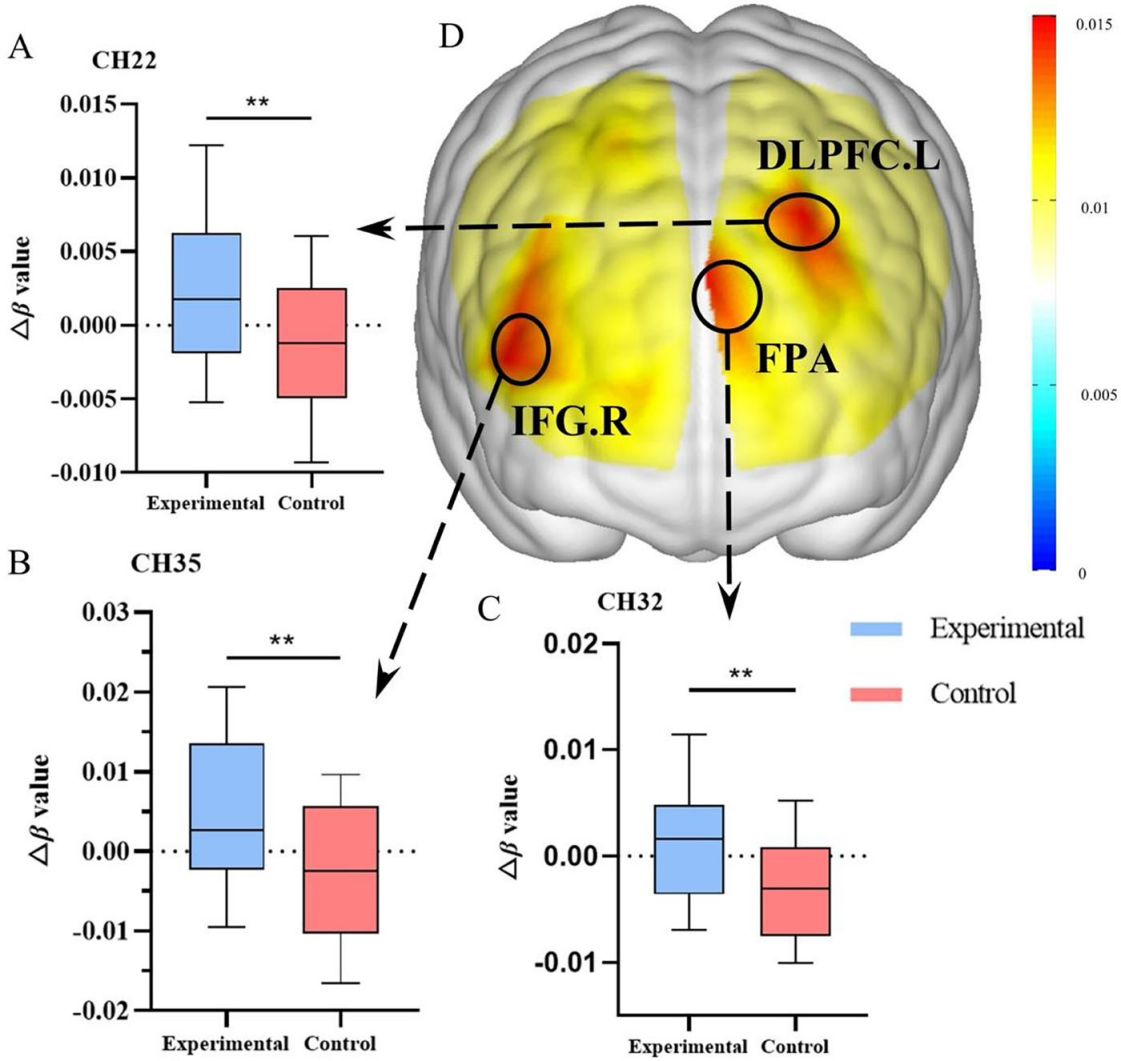
**Changes in cortical activation during dual‐task performance in both groups**. (A) Comparison of the difference in *β* values of channel 22 activation before and after intervention in both groups; (B) Comparison of the difference in *β* values of channel 35 activation before and after intervention in both groups; (C) Comparison of the difference in *β* values of channel 32 activation before and after intervention in both groups; (D) Changes in activation before and after intervention under cognitive walking dual‐task conditions for both groups, with circles indicating regions where differences in activation changes before and after intervention were observed in the dual‐task cognitive walking state. The boxplots show individual changes in task‐related *β* values (post‐intervention minus baseline). Statistical significance indicates between‐group differences in change and does not represent a within‐group test of activation change. The error bars represent the 95% CIs. The fNIRS results are shown at the *p* < 0.05 level and were corrected for FDR. *p < 0.05, **p < 0.01, ***p < 0.001; R, right; L, left; pre‐test, preintervention testing; post‐test, post‐intervention testing; *Δ*, post‐intervention minus preintervention. FPA, frontal pole area.

After 6 weeks of intervention, the changes in activation values for channels CH22 (located at DLPFC.L) (*p* = 0.003), CH32 (located at FPA) (*p* = 0.003), and CH35 (located at IFG.R) (*p* = 0.004) in the experimental group were significantly greater than those observed in the control group (Figure 5) (uncorrected *p* < 0.01, FDR‐corrected *p* < 0.05). There were no significant differences in the *β* values of the remaining channels between the experimental and control groups (Tables S4–S6).

### 3.6 Functional Connectivity

**FIGURE 6:**
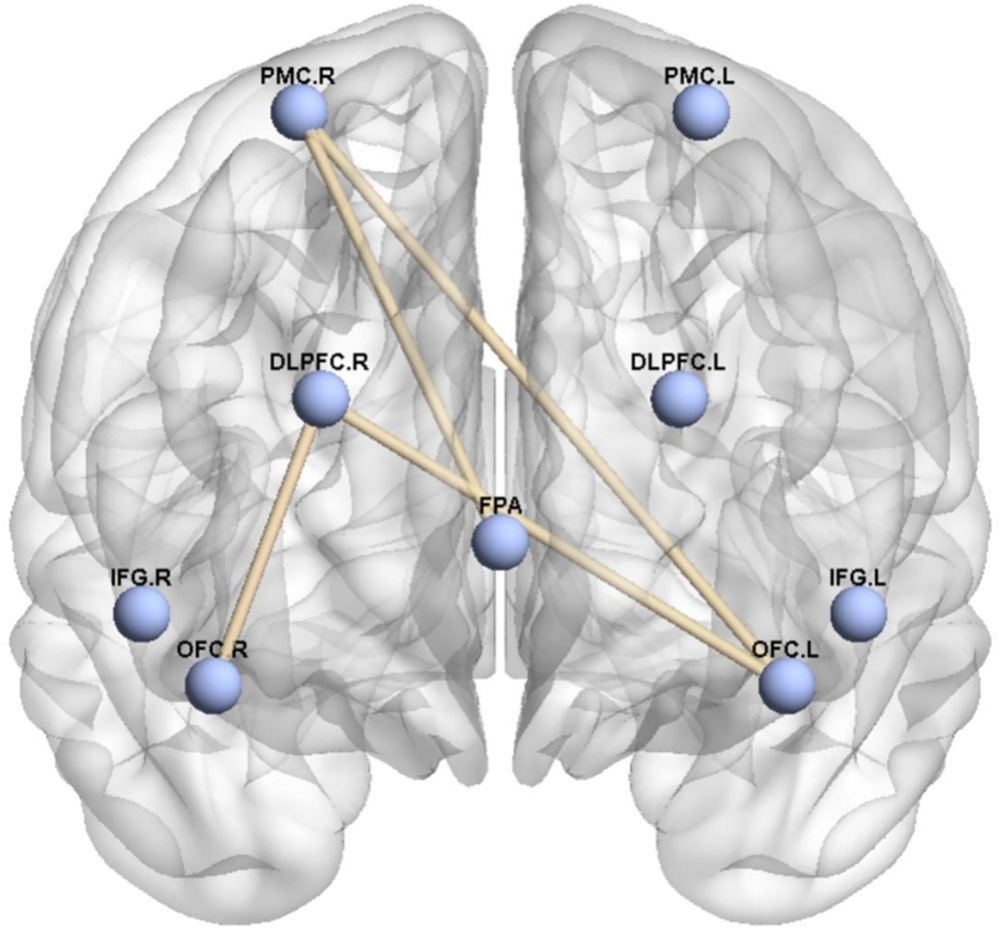
**Four pairs of functional connectivity strength changes in the experimental group were significantly greater than those in the control group**. DLPFC, dorsolateral prefrontal cortex; FPA, frontal pole area; IFG, inferior frontal gyrus; OFC, orbitofrontal cortex; PMC, premotor cortex. R, right; L, left; the fNIRS results are shown at the *p* < 0.05 level and were corrected for FDR.

After 6 weeks of intervention, the changes in functional connectivity strength for the PMC.R‐FPA (CH1‐CH38) (*p* = 0.000492), PMC.R‐OFC.L (CH1‐CH44)(*p* = 0.001), DLPFC.R‐OFC.R (CH26‐CH41)(*p* = 0.000276), and DLPFC.R‐OFC.L (CH26‐CH44)(*p* = 0.001)in the experimental group were significantly greater than those observed in the control group (Figure 6) (uncorrected *p* < 0.01, FDR‐corrected *p* < 0.05). There were no significant differences in the functional connectivity strength between the remaining channel pairs of the experimental and control groups (Tables S7–S9).

### 3.7 Correlation Analysis

**FIGURE 7:**
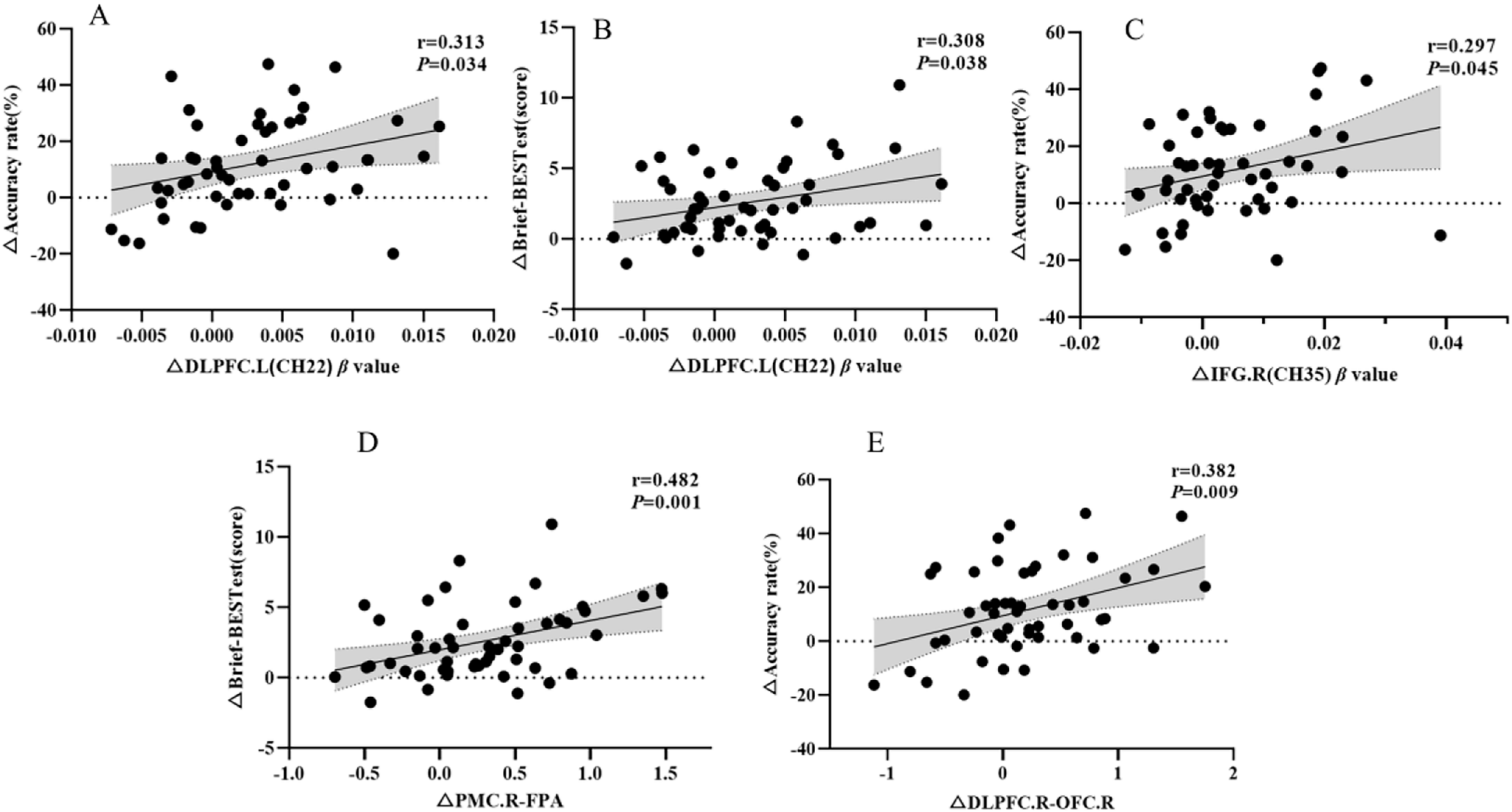
**Correlations between changes in cerebral hemodynamics and improvements in behavior in the experimental group**. (A) The correlation between changes in *β* values for channel 22 (*Δ* CH22 *β* value) and changes in the dual‐task accuracy rate (*Δ* accuracy rate). (B) The correlation between changes in *β* values for channel 22 (*Δ* CH22 *β* value) and changes in the Brief‐BESTest scores (*Δ* Brief‐BESTest). (C) The correlation between changes in *β* values for channel 35 (*Δ* CH35 *β* value) and changes in the dual‐task accuracy rate (*Δ* accuracy rate). (D) The correlation between the functional connectivity change value (*Δ* correlation CH1–CH38) and changes in the Brief‐BESTest scores (*Δ* Brief‐BESTest). (E) The correlation between the functional connectivity change value (*Δ* correlation CH26–CH41) and changes in the dual‐task accuracy rate (*Δ* accuracy rate); *Δ*, post‐intervention minus preintervention; CH, channel. Brief‐BESTest, brief balance evaluation systems test; FPA, frontal pole area.

The results of the correlation analysis indicated that the level of brain activation was associated with changes in functional connectivity and improvements in behavioral performance (Figure 7). Specifically, changes in CH22 (located in the DLPFC.L) *β* value were positively correlated with dual‐task accuracy (*r* = 0.313, *p* = 0.034) and the Brief‐BESTest scores (*r* = 0.308, *p* = 0.038). Changes in CH35 (located in the IFG.R) *β* value were positively correlated with dual‐task accuracy (*r* = 0.297, *p* = 0.045). Changes in PMC.R–FPA connectivity (CH1–CH38) were positively associated with changes in Brief‐BESTest scores (*r* = 0.482, *p* = 0.001), and changes in DLPFC.R–OFC.R connectivity (CH26–CH41) were positively associated with changes in serial‐7 subtraction accuracy (*r* = 0.382, *p* = 0.009) (Table S10).

## 4 Discussion

Using fNIRS, we measured cerebral hemodynamic changes in the prefrontal and motor areas during dual‐task performance. Our findings suggest that altered activation and functional connectivity in these regions may explain why active rTMS combined with Tai Chi Chuan provided additional benefits for attentional allocation in older adults with sleep disorders and MCI. These findings align with our hypothesis, indicating that 1‐Hz rTMS targeting the right DLPFC was more effective than sham rTMS was in enhancing the benefits of Tai Chi for dual‐task performance in this population. Furthermore, the improvements in dual‐task performance were associated with increased activation and increased functional connectivity in the prefrontal and premotor cortex.

### 4.1 Additional Effects of rTMS Combined With Tai Chi Chuan on Dual‐Task Performance and Gait Balance

The greater gains in dual‐task performance and balance in the experimental group are consistent with evidence that rTMS can augment exercise‐based cognitive‐motor interventions in older adults (Chung et al. 2020). Cognitive resource theory posits that concurrent motor and cognitive tasks compete for a finite pool of attentional resources (Panichello and Buschman 2021). Successful dual‐task performance therefore requires these resources to be flexibly distributed between motor and cognitive demands (Kaping et al. 2011). Executive and working‐memory deficits may further restrict this capacity in older adults with sleep disorders and MCI (Cassidy‐Eagle et al. 2018). Impaired brain clearance may provide one biological link between sleep disturbance and reduced cognitive‐motor function. Sleep disruption may hinder the removal of neurotoxic metabolites and disturb neuronal function and interregional communication (Jiang‐Xie et al. 2024; Xie et al. 2013). Such disruption could further compromise executive control and attentional allocation during dual‐task performance.

Tai Chi Chuan may strengthen cognitive‐motor coordination by simultaneously engaging motor and cognitive processes (Chen et al. 2022). Because both groups completed the same standardized Tai Chi Chuan program, the between‐group comparison estimates the effect of active versus sham rTMS within a common Tai Chi Chuan context. However, the absence of a Tai Chi Chuan–only group, an rTMS‐only group, and a non‐exercise control group means that the independent effects of Tai Chi Chuan and rTMS, as well as their potential interaction, cannot be determined. Likewise, pre–post changes in the sham‐rTMS group cannot be attributed specifically to Tai Chi Chuan. The observed between‐group differences should therefore be interpreted as the additional effect of active rTMS in the context of a shared Tai Chi Chuan program. Notably, the experimental group also showed a greater reduction in PSQI scores than the control group, indicating a greater improvement in subjective sleep quality. In 5xFAD mice, rTMS enhanced glymphatic and meningeal lymphatic drainage, reduced amyloid‐β accumulation, and preserved memory performance (Lin et al. 2021). In patients with chronic insomnia, sleep improvements after rTMS coincided with changes in the diffusion tensor image analysis along the perivascular space (DTI‐ALPS) index (Gao et al. 2025). This index is an indirect imaging marker of glymphatic function. Together, these findings raise the possibility that active rTMS partly supports sleep‐related brain clearance processes, which may help preserve the executive resources required for dual‐task control.

### 4.2 Hemodynamic Characteristics Underlying the Additional Effects of rTMS Combined With Tai Chi Chuan on Dual‐Task Performance

After 6 weeks of intervention, the experimental group showed significantly higher left DLPFC *β* values than the control group. Changes in left DLPFC *β* values were positively associated with changes in dual‐task subtraction accuracy and Brief‐BESTest scores. These findings suggest that active rTMS, when combined with Tai Chi Chuan, was associated with greater left DLPFC recruitment during dual‐task performance. This recruitment may have supported attentional allocation and balance performance, although the observed associations do not establish causality.

This pattern is consistent with previous fNIRS evidence of rTMS‐related improvements in neural efficiency in adults (Curtin et al. 2019). The DLPFC contributes to attentional allocation and the processing of task‐relevant information. Greater activation in this region may therefore reflect increased recruitment of cognitive resources during task performance (Smith et al. 2019; Yang et al. 2024). Although 1‐Hz rTMS is conventionally described as inhibitory, this characterization is based primarily on transient changes in corticospinal excitability, typically assessed using motor‐evoked potentials following stimulation of the motor cortex (Fitzgerald et al. 2006). It therefore cannot be directly extrapolated to task‐evoked hemodynamic activity or functional connectivity after repeated DLPFC stimulation. Notably, in older adults, 1‐Hz rTMS applied to the left DLPFC reduced local activity while eliciting a more distributed pattern of bilateral prefrontal connectivity during successful memory encoding (Davis et al. 2017). This finding suggests that reduced local activity may coexist with increased communication across distributed prefrontal networks. In fNIRS, functional connectivity is estimated from the temporal synchronization of hemodynamic signals between spatially separated regions and does not provide a direct measure of cortical excitability (Niu and He 2014). As cortical excitability at the stimulated right DLPFC was not measured in the present study, we cannot determine whether local inhibition occurred or whether it caused the observed connectivity changes. The increased prefrontal and premotor connectivity should therefore be interpreted as a potential network‐level reorganization following repeated active rTMS, rather than as evidence of increased local excitability at the stimulation site. Although low‐frequency stimulation targeted the right DLPFC, the experimental group showed greater activation in the left DLPFC. This lateralized response is consistent with possible transhemispheric modulation following low‐frequency stimulation (Chen et al. 2024). The observed response may also help interpret our previous finding that low‐frequency right‐DLPFC rTMS augmented the effects of Tai Chi Chuan on sleep quality and cognitive function (Liu et al. 2025). Contralateral prefrontal recruitment may contribute to the additional effects of rTMS on brain activation and functional connectivity. Previous interventions targeting cognition in older adults have predominantly used high‐frequency rTMS protocols (Chu et al. 2021). Our findings suggest that low‐frequency stimulation may also support cognitive functions such as attentional allocation. The contralateral activation observed here offers one possible explanation, but it does not establish a unique mechanism.

The experimental group also showed a significantly greater increase in functional connectivity between the right DLPFC and OFC than the control group. This connectivity change was positively associated with improvement in dual‐task subtraction accuracy. Because both groups received the same Tai Chi Chuan program, the between‐group difference may reflect an additional effect of active rather than sham rTMS. The OFC contributes to value assessment, decision‐making, and the suppression of task‐irrelevant information (Fan et al. 2024). Stronger OFC‐DLPFC connectivity may reflect closer coordination between attentional selection and executive control (Jenkins et al. 2021). Such coordination could support more flexible allocation of attentional resources and reduce interference between concurrent tasks (Lin et al. 2020). This interpretation is consistent with the association between connectivity changes and dual‐task subtraction accuracy.

Attentional allocation during dual‐task performance engages several frontal regions, including the FPA and IFG (Tremblay et al. 2015). After 6 weeks, the experimental group showed greater activation in the FPA and right IFG than the control group during dual‐task performance. Changes in right inferior frontal activation were positively associated with changes in dual‐task accuracy. The concurrent recruitment of these regions is consistent with their shared involvement in cognitive control (Muhle‐Karbe et al. 2016; Santinelli et al. 2024). However, regional activation alone cannot confirm functional cooperation among these areas.

After 6 weeks of intervention, the experimental group also showed greater functional connectivity between the right PMC and FPA than the control group. Changes in this connectivity were positively associated with improvements in Brief‐BESTest scores. This pattern suggests that active rTMS may have strengthened premotor‐prefrontal coupling, which was associated with improved balance performance. The PMC contributes to translating prefrontal goals into motor programs and integrating proprioceptive information relevant to walking (Butler et al. 2017). More efficient fronto‐motor coordination may reduce the cognitive demands of gait, leaving more resources available for concurrent cognitive tasks (Rémy et al. 2010). These findings identify premotor–prefrontal connectivity as a neural correlate of improved balance performance following active rTMS combined with Tai Chi Chuan.

### 4.3 Limitations

This study has several limitations. First, the two‐arm design did not include a Tai Chi Chuan–only group, an rTMS‐only group, or a non‐exercise control group. Although the design allowed us to estimate the add‐on effect of active rTMS within a common Tai Chi Chuan program, it could not determine the independent effects of Tai Chi Chuan and rTMS or their potential interaction. Second, although sham rTMS, matched intervention schedules, standardized Tai Chi supervision, and blinded outcome assessment were used to reduce placebo, extra‐attention, and expectation effects, residual expectancy effects cannot be excluded. Third, the dose–response relationship and mechanisms of different rTMS parameters, including intensity, frequency, and duration, remain unclear. We did not directly quantify task prioritization or the allocation of attention between walking and subtraction. Therefore, although equal task priority was standardized through instructions, individual differences in prioritization strategy cannot be excluded. Future studies should optimize these parameters to refine the combined intervention protocol. Fourth, fNIRS was limited to the prefrontal and premotor cortices. Future work should expand cortical coverage and analytical approaches to better characterize the neural effects of combined rTMS and Tai Chi Chuan interventions.

## 5 Conclusion

In older adults with sleep disorders and MCI, the experimental group receiving 1‐Hz rTMS plus Tai Chi Chuan showed greater improvements in dual‐task performance and balance than the control group. fNIRS analyses showed greater left DLPFC activation and stronger functional connectivity between the right DLPFC and OFC and between the right PMC and frontal pole. These hemodynamic changes were associated with improvements in dual‐task accuracy and Brief‐BESTest scores and may reflect the modulation of prefrontal and fronto‐motor networks. The findings support combined rTMS and Tai Chi Chuan as a potential strategy for improving cognitive‐motor function in this population.

## Author Contributions

**Zhenxing Guo**: investigation, data curation, formal analysis, visualization, writing – original draft. **Kaifeng Xu**: investigation, data curation, formal analysis, writing – review and editing. **Linxin Bai**: investigation, data curation. **Yuxuan Sun**: investigation, data curation. **Lin Zhang**: investigation, data curation. **Jiahui Gao**: investigation, funding acquisition, supervision. **Lidian Chen**: conceptualization, resources, supervision. **Zhizhen Liu**: conceptualization, methodology, funding acquisition, supervision.

## Funding

This study was supported by Fujian Provincial Natural Science Foundation [grant number 2024Y9531], Special Fund of Provincial Units for Education and Scientific Research in 2022 [great number X2022004], Natural Science Foundation for Distinguished Young Scholars of Fujian Province of China [grant number 2022J06028], Scientific Research Foundation for the top youth talents of Fujian University of Traditional Chinese Medicine [grant number XQC2023005], and Science and Technology Major Project of Fujian University of Traditional Chinese Medicine [grant number XJB2022007].

## Ethics Statement

This study was approved by the ethics committee of the Affiliated Rehabilitation Hospital of Fujian University of Traditional Chinese Medicine (approval number: 2022KY‐024‐01). The study is registered with the Chinese Clinical Trial Registry (ChiCTR2200063274).

## Consent

All subjects signed a written informed consent form before initiating the trial.

## Conflicts of Interest

The authors declare no conflicts of interest.

## Supporting information




**Supplementary Materials**: brb371743‐sup‐0001‐SuppMat.docx

**Supplementary Materials**: brb371743‐sup‐0002‐SuppMat.docx

**Supplementary Materials**: brb371743‐sup‐0003‐SuppMat.docx

## Data Availability

The data will be made available from the authors on the basis of reasonable requests. For detailed information, please see Supporting Information 3.
