# Supplementary material for: Additional Effects of 1‐Hz rTMS Combined With Tai Chi Chuan on Dual‐Task Performance Among Older Adults With Sleep Disorders and MCI: An fNIRS‐Based Randomized Controlled Trial
Source: Brain Behav. 2026 Sep 14;16(9):e71743. doi: 10.1002/brb3.71743 (PMC13573082; doi:10.1002/brb3.71743)
Supplement: Supplementary file 1 [file BRB3-16-e71743-s001.docx]

**Table S3. Comparison of Dual-Task Performance and gait balance ability of participants in the two groups**.

| **Outcomes** |  | **Experimental** | **Sham** | **Z** | ***P*** |
| --- | --- | --- | --- | --- | --- |
| **Total responses,number** | pre-text | 20(16-27) | 19(14-22) | -2.136 | 0.033 |
|  | post-text | 21(16-28) | 18(14-25) | -2.272 | 0.023 |
|  | △ | 0(-3-3) | -1(-3-4) | -0.397 | 0.692 |
| **Correct responses,number** | pre-text | 17(10-25) | 15(8-19) | -1.536 | 0.125 |
|  | post-text | 18(14-28) | 15(9-19) | -3.268 | 0.001 |
|  | △ | 3(-1-6) | 0(-3-5) | -2.078 | 0.038 |
| **Accuracy rate, %** | pre-text | 85.71(68.75-92.86) | 83.28(66.67-93.33) | -0.575 | 0.565 |
|  | post-text | 94.44(85-100) | 82.57(68.71-89.36) | -4.444 | ＜0.001 |
|  | △ | 7.41(0-23.02) | -1.70(-11.66-10.41) | -2.79 | 0.005 |
| **Brief-BESTest, score** | pre-text | 21(18-22) | 20(17-23) | -0.996 | 0.319 |
|  | post-text | 23(22-24) | 22(18-23) | -4.404 | ＜0.001 |
|  | △ | 2(1-5) | 1(0-4) | -2.309 | 0.021 |

The values are reported as medians (interquartile range).

pre-text, baseline testing; post-text, 6 weeks post-intervention testing; Δ, 6 weeks post-intervention testing minus baseline testing

**Table S4. Comparison of *β* values in the baseline between the two groups.**

| **Brain regions** | **Channels** | **Experimental** | **Sham** | **Z** | ***P*** |
| --- | --- | --- | --- | --- | --- |
| PMC | CH1 | 0.00378(-0.00337-0.00833) | 0.00193(-0.00264-0.00728) | -0.201 | 0.841 |
| PMC | CH3 | 0.00263(-0.00499-0.00781) | 0.0043(-0.00028-0.00934) | 1.652 | 0.098 |
| PMC | CH4 | 0.00065(-0.00245-0.00755) | 0.00239(-0.00203-0.00591) | 0.551 | 0.582 |
| PMC | CH7 | 0.00213(-0.00093-0.00914) | 0.00097(-0.00177-0.00473) | -1.797 | 0.072 |
| DLPFC | CH20 | 0.00079(-0.00149-0.00533) | 0.00205(-0.00105-0.00537) | 0.653 | 0.514 |
| DLPFC | CH22 | 0.0019(-0.00049-0.00515) | 0.00165(-0.00014-0.00641) | 0.541 | 0.589 |
| DLPFC | CH26 | 0.00167(-0.0009-0.00543) | 0.00297(-0.00077-0.0063) | 0.867 | 0.386 |
| DLPFC | CH27 | 0.0019(0.00006-0.00537) | 0.00176(-0.00107-0.00459) | -0.185 | 0.853 |
| FPA | CH32 | 0.00075(-0.00161-0.00501) | 0.00294(0.00027-0.00568) | 1.952 | 0.051 |
| IFG | CH35 | 0.0041(0.00003-0.00877) | 0.00845(0.00347-0.01369) | 2.295 | 0.054 |
| IFG | CH36 | 0.00426(-0.00042-0.00836) | 0.00434(-0.00147-0.00844) | 0.135 | 0.892 |
| FPA | CH37 | 0.00249(-0.00021-0.00593) | 0.00247(-0.00181-0.0077) | -0.379 | 0.704 |
| FPA | CH38 | 0.00214(-0.00059-0.00555) | 0.00349(-0.00243-0.00724) | 0.267 | 0.789 |
| IFG | CH39 | 0.00413(-0.00032-0.00821) | 0.00533(-0.0007-0.00877) | 0.452 | 0.651 |
| IFG | CH40 | 0.00621(0.00187-0.01139) | 0.00451(-0.00047-0.00887) | -1.135 | 0.257 |
| OFC | CH41 | 0.00233(-0.00223-0.00813) | 0.00552(-0.00007-0.00907) | 1.349 | 0.177 |
| OFC | CH42 | 0.0032(-0.00142-0.00765) | 0.00435(-0.00237-0.00715) | 0.195 | 0.846 |
| OFC | CH44 | 0.00313(-0.0016-0.00754) | 0.00478(-0.0014-0.00858) | 0.696 | 0.486 |
| OFC | CH45 | 0.00412(-0.00269-0.00833) | 0.00366(0.00041-0.01101) | 0.920 | 0.357 |

The values are reported as medians (interquartile range).

PMC,premotor area;DLPFC,dorsolateral prefrontal cortex;FPA,frontal pole area; IFG, inferior frontal gyrus; OFC,.orbitofrontal cortex;CH,channel

**Table S5. Comparison of *β* values in 6 weeks post-intervention between the two groups.**

| **Brain regions** | **Channels** | **Experimental** | **Sham** | **Z** | ***P*** | **FDR** |
| --- | --- | --- | --- | --- | --- | --- |
| PMC | CH1 | 0.00369(-0.00357-0.0098) | 0.00121(-0.00281-0.00616) | -0.838 | 0.402 | 0.486 |
| PMC | CH3 | 0.00448(-0.00619-0.01226) | 0.00681(-0.00051-0.01167) | 0.825 | 0.41 | 0.486 |
| PMC | CH4 | 0.00484(-0.00106-0.00983) | 0.00103(-0.00265-0.00762) | -1.471 | 0.141 | 0.243 |
| PMC | CH7 | 0.00384(-0.00027-0.01097) | 0.00154(-0.0043-0.00917) | -1.332 | 0.183 | 0.289 |
| DLPFC | CH20 | 0.00386(0.00081-0.0063) | 0.00408(-0.00126-0.00685) | 0.165 | 0.869 | 0.869 |
| **DLPFC** | **CH22** | **0.00463(0.00028-0.00737)** | **0.00158(-0.00126-0.00348)** | **-3.225** | **0.001** | **0.019** |
| DLPFC | CH26 | 0.00238(-0.00122-0.00677) | 0.00249(-0.00088-0.00541) | -0.297 | 0.767 | 0.809 |
| DLPFC | CH27 | 0.00314(-0.00035-0.00615) | 0.00272(-0.00195-0.00692) | -0.396 | 0.692 | 0.773 |
| FPA | CH32 | 0.00075(-0.00161-0.00501) | 0.00294(0.00027-0.00568) | 1.952 | 0.051 | 0.118 |
| IFG | CH35 | 0.00699(0.00332-0.01663) | 0.00588(-0.00011-0.01054) | -1.656 | 0.098 | 0.186 |
| IFG | CH36 | 0.00443(0.00124-0.01121) | 0.00238(-0.00174-0.00713) | -1.913 | 0.056 | 0.118 |
| FPA | CH37 | 0.00432(0.00059-0.00885) | 0.0008(-0.00256-0.00638) | -2.157 | 0.031 | 0.098 |
| FPA | CH38 | 0.0041(-0.00078-0.00709) | 0.00076(-0.00339-0.00453) | -2.328 | 0.02 | 0.098 |
| **IFG** | **CH39** | **0.00633(-0.00021-0.01012)** | **0.00234(-0.00321-0.00773)** | **-2.843** | **0.004** | **0.038** |
| IFG | CH40 | 0.0079(0.00128-0.01511) | 0.00444(0.00021-0.00838) | 2.256 | 0.024 | 0.098 |
| OFC | CH41 | 0.00568(-0.00188-0.01179) | 0.00225(-0.0011-0.00821) | -1.2 | 0.23 | 0.336 |
| OFC | CH42 | 0.00483(0.00128-0.00931) | 0.00236(-0.00069-0.0065) | -2.229 | 0.026 | 0.098 |
| OFC | CH44 | 0.00396(-0.00011-0.00892) | 0.00179(-0.00225-0.00661) | -1.946 | 0.052 | 0.118 |
| OFC | CH45 | 0.00573(0.00085-0.01227) | 0.00488(0.00015-0.00794) | -1.069 | 0.285 | 0.386 |

The values are reported as medians (interquartile range).

PMC,premotor area;DLPFC,dorsolateral prefrontal cortex;FPA,frontal pole area; IFG, inferior frontal gyrus; OFC,.orbitofrontal cortex;CH,channel

**Table S6. Comparison of *β* change values(△) between the two groups**

| **Brain regions** | **Channels** | **Experimental** | **Sham** | **Z** | ***P*** | **FDR** |
| --- | --- | --- | --- | --- | --- | --- |
| PMC | CH1 | -0.00187(-0.00495-0.00701) | -0.00173(-0.00611-0.00575) | -0.712 | 0.476 | 0.646 |
| PMC | CH3 | -0.00046(-0.00825-0.0101) | 0.00042(-0.0054-0.00971) | 0.528 | 0.598 | 0.707 |
| PMC | CH4 | 0.0045(-0.0037-0.0108) | 0.00012(-0.00531-0.00628) | -1.385 | 0.166 | 0.287 |
| PMC | CH7 | 0.00109(-0.00569-0.00851) | -0.00019(-0.00549-0.01329) | 0.429 | 0.668 | 0.707 |
| DLPFC | CH20 | 0.00213(-0.00113-0.00558) | 0.00066(-0.00599-0.00709) | -0.699 | 0.484 | 0.646 |
| **DLPFC** | **CH22** | **0.00174(-0.00189-0.00624)** | **-0.00125(-0.00526-0.00242)** | **-3.021** | **0.003** | **0.025** |
| DLPFC | CH26 | 0.00204(-0.00365-0.00492) | -0.00043(-0.00608-0.00359) | -1.537 | 0.124 | 0.262 |
| DLPFC | CH27 | 0.00088(-0.0024-0.00417) | 0.00066(-0.00365-0.00562) | -0.376 | 0.707 | 0.707 |
| **FPA** | **CH32** | **0.00165(-0.00357-0.0048)** | **-0.00309(-0.00733-0.00075)** | **-2.935** | **0.003** | **0.025** |
| **IFG** | **CH35** | **0.00261(-0.00227-0.01351)** | **-0.00252(-0.01022-0.00561)** | **-2.843** | **0.004** | **0.025** |
| IFG | CH36 | 0.00004(-0.00478-0.00586) | 0.00024(-0.00876-0.006) | -0.660 | 0.51 | 0.646 |
| FPA | CH37 | 0.00035(-0.00374-0.00506) | -0.00139(-0.00579-0.00586) | -1.088 | 0.276 | 0.437 |
| FPA | CH38 | 0.00129(-0.00499-0.00441) | -0.00179(-0.0077-0.00281) | -1.609 | 0.108 | 0.257 |
| IFG | CH39 | 0.00166(-0.00526-0.0087) | -0.00348(-0.00874-0.00217) | -2.401 | 0.016 | 0.076 |
| IFG | CH40 | -0.00067(-0.00566-0.00923) | 0.00054(-0.00594-0.00462) | -0.416 | 0.678 | 0.707 |
| OFC | CH41 | 0.0017(-0.00876-0.00865) | -0.00075(-0.00857-0.00368) | -1.431 | 0.152 | 0.287 |
| OFC | CH42 | 0.00118(-0.00405-0.00705) | -0.00196(-0.00849-0.00624) | -1.662 | 0.096 | 0.257 |
| OFC | CH44 | 0.0011(-0.00489-0.00659) | -0.00097(-0.00903-0.00444) | -1.985 | 0.047 | 0.179 |
| OFC | CH45 | 0.00021(-0.0027-0.00847) | -0.00174(-0.01041-0.00469) | -1.774 | 0.076 | 0.241 |

The values are reported as medians (interquartile range).

PMC,premotor area;DLPFC,dorsolateral prefrontal cortex;FPA,frontal pole area; IFG,inferior frontal gyrus; OFC,.orbitofrontal cortex;CH,channel；Δ, 6 weeks post-intervention testing minus baseline testing

**Table S7. Comparison of functional connectivity strength in the baseline between the two groups**

| **Brain regions** | **Channel pairs** | **Experimental** | **Sham** | **t** | ***P*** | **FDR** |
| --- | --- | --- | --- | --- | --- | --- |
| PMC.R-PMC.L | (1,3) | 0.586±0.369 | 0.562±0.368 | 0.333 | 0.739 | 0.989282208588957 |
| PMC.R-PMC.R | (1,4) | 0.296±0.426 | 0.337±0.455 | -0.469 | 0.64 | 0.989282208588957 |
| PMC.R-PMC.L | (1,7) | 0.393±0.43 | 0.4±0.423 | -0.073 | 0.942 | 0.989282208588957 |
| PMC.R-DLPFC.R | (1,20) | 0.383±0.455 | 0.306±0.462 | 0.859 | 0.392 | 0.989282208588957 |
| PMC.R-DLPFC.L | (1,22) | 0.322±0.444 | 0.381±0.444 | -0.672 | 0.503 | 0.989282208588957 |
| PMC.R-DLPFC.R | (1,26) | 0.371±0.433 | 0.394±0.439 | -0.27 | 0.788 | 0.989282208588957 |
| PMC.R-DLPFC.L | (1,27) | 0.41±0.447 | 0.384±0.444 | 0.292 | 0.771 | 0.989282208588957 |
| PMC.R-FPA | (1,32) | 0.299±0.544 | 0.272±0.475 | 0.266 | 0.791 | 0.989282208588957 |
| PMC.R-IFG.R | (1,35) | 0.39±0.432 | 0.305±0.48 | 0.942 | 0.348 | 0.989282208588957 |
| PMC.R-IFG.R | (1,36) | 0.269±0.494 | 0.312±0.447 | -0.465 | 0.643 | 0.989282208588957 |
| PMC.R-FPA | (1,37) | 0.389±0.402 | 0.324±0.388 | 0.844 | 0.4 | 0.989282208588957 |
| PMC.R-FPA | (1,38) | 0.116±0.511 | 0.285±0.481 | -1.737 | 0.085 | 0.989282208588957 |
| PMC.R-IFG.L | (1,39) | 0.204±0.457 | 0.282±0.433 | -0.89 | 0.376 | 0.989282208588957 |
| PMC.R-IFG.L | (1,40) | 0.305±0.451 | 0.319±0.414 | -0.166 | 0.868 | 0.989282208588957 |
| PMC.R-OFC.R | (1,41) | 0.279±0.461 | 0.318±0.453 | -0.43 | 0.668 | 0.989282208588957 |
| PMC.R-OFC.R | (1,42) | 0.19±0.52 | 0.168±0.446 | 0.235 | 0.814 | 0.989282208588957 |
| PMC.R-OFC.L | (1,44) | 0.182±0.476 | 0.307±0.45 | -1.365 | 0.175 | 0.989282208588957 |
| PMC.R-OFC.L | (1,45) | 0.187±0.542 | 0.298±0.416 | -1.161 | 0.248 | 0.989282208588957 |
| PMC.R-PMC.L | (3,4) | 0.342±0.446 | 0.366±0.448 | -0.271 | 0.787 | 0.989282208588957 |
| PMC.L-PMC.L | (3,7) | 0.414±0.467 | 0.32±0.461 | 1.03 | 0.306 | 0.989282208588957 |
| PMC.L-DLPFC.R | (3,20) | 0.441±0.512 | 0.369±0.489 | 0.724 | 0.471 | 0.989282208588957 |
| PMC.L-DLPFC.L | (3,22) | 0.397±0.495 | 0.343±0.476 | 0.567 | 0.572 | 0.989282208588957 |
| PMC.L-DLPFC.R | (3,26) | 0.475±0.429 | 0.394±0.437 | 0.95 | 0.344 | 0.989282208588957 |
| PMC.L-DLPFC.L | (3,27) | 0.466±0.42 | 0.318±0.483 | 1.669 | 0.098 | 0.989282208588957 |
| PMC.L-FPA | (3,32) | 0.342±0.583 | 0.347±0.445 | -0.042 | 0.967 | 0.993233532934132 |
| PMC.L-IFG.R | (3,35) | 0.46±0.46 | 0.227±0.496 | 2.463 | 0.015 | 0.989282208588957 |
| PMC.L-IFG.R | (3,36) | 0.353±0.515 | 0.273±0.503 | 0.797 | 0.427 | 0.989282208588957 |
| PMC.L-FPA | (3,37) | 0.376±0.488 | 0.225±0.474 | 1.596 | 0.114 | 0.989282208588957 |
| PMC.L-FPA | (3,38) | 0.127±0.583 | 0.104±0.536 | 0.202 | 0.84 | 0.989282208588957 |
| PMC.L-IFG.L | (3,39) | 0.241±0.5 | 0.161±0.47 | 0.835 | 0.406 | 0.989282208588957 |
| PMC.L-IFG.L | (3,40) | 0.393±0.462 | 0.222±0.49 | 1.819 | 0.072 | 0.989282208588957 |
| PMC.R-PMC.R | (3,41) | 0.252±0.524 | 0.364±0.425 | -1.198 | 0.234 | 0.989282208588957 |
| PMC.R-PMC.R | (3,42) | 0.21±0.593 | 0.153±0.484 | 0.54 | 0.591 | 0.989282208588957 |
| PMC.R-PMC.L | (3,44) | 0.186±0.565 | 0.284±0.488 | -0.94 | 0.349 | 0.989282208588957 |
| PMC.R-PMC.L | (3,45) | 0.232±0.594 | 0.222±0.513 | 0.093 | 0.926 | 0.989282208588957 |
| PMC.R-PMC.L | (4,7) | 0.338±0.383 | 0.297±0.463 | 0.482 | 0.631 | 0.989282208588957 |
| PMC.R-DLPFC.R | (4,20) | 0.276±0.474 | 0.268±0.467 | 0.092 | 0.927 | 0.989282208588957 |
| PMC.R-DLPFC.L | (4,22) | 0.259±0.49 | 0.302±0.443 | -0.473 | 0.637 | 0.989282208588957 |
| PMC.R-DLPFC.R | (4,26) | 0.347±0.4 | 0.337±0.435 | 0.119 | 0.906 | 0.989282208588957 |
| PMC.R-DLPFC.L | (4,27) | 0.309±0.456 | 0.309±0.447 | 0.008 | 0.994 | 0.994 |
| PMC.R-FPA | (4,32) | 0.244±0.443 | 0.306±0.451 | -0.702 | 0.484 | 0.989282208588957 |
| PMC.R-IFG.R | (4,35) | 0.269±0.449 | 0.304±0.436 | -0.405 | 0.687 | 0.989282208588957 |
| PMC.R-IFG.R | (4,36) | 0.296±0.445 | 0.218±0.495 | 0.846 | 0.4 | 0.989282208588957 |
| PMC.R-FPA | (4,37) | 0.305±0.419 | 0.186±0.443 | 1.392 | 0.167 | 0.989282208588957 |
| PMC.R-FPA | (4,38) | 0.209±0.464 | 0.091±0.498 | 1.252 | 0.213 | 0.989282208588957 |
| PMC.R-IFG.L | (4,39) | 0.147±0.441 | 0.149±0.439 | -0.016 | 0.988 | 0.994 |
| PMC.R-IFG.L | (4,40) | 0.242±0.451 | 0.199±0.473 | 0.473 | 0.637 | 0.989282208588957 |
| PMC.R-OFC.R | (4,41) | 0.271±0.463 | 0.26±0.484 | 0.119 | 0.905 | 0.989282208588957 |
| PMC.R-OFC.R | (4,42) | 0.247±0.467 | 0.106±0.482 | 1.515 | 0.133 | 0.989282208588957 |
| PMC.R-OFC.L | (4,44) | 0.249±0.453 | 0.2±0.492 | 0.523 | 0.602 | 0.989282208588957 |
| PMC.R-OFC.L | (4,45) | 0.181±0.461 | 0.146±0.485 | 0.37 | 0.712 | 0.989282208588957 |
| PMC.L-DLPFC.R | (7,20) | 0.368±0.504 | 0.398±0.482 | -0.308 | 0.759 | 0.989282208588957 |
| PMC.L-DLPFC.L | (7,22) | 0.319±0.493 | 0.411±0.444 | -0.995 | 0.322 | 0.989282208588957 |
| PMC.L-DLPFC.R | (7,26) | 0.38±0.467 | 0.429±0.443 | -0.548 | 0.585 | 0.989282208588957 |
| PMC.L-DLPFC.L | (7,27) | 0.383±0.478 | 0.489±0.449 | -1.161 | 0.248 | 0.989282208588957 |
| PMC.L-FPA | (7,32) | 0.312±0.536 | 0.332±0.514 | -0.193 | 0.848 | 0.989282208588957 |
| PMC.L-IFG.R | (7,35) | 0.331±0.486 | 0.28±0.486 | 0.533 | 0.595 | 0.989282208588957 |
| PMC.L-IFG.R | (7,36) | 0.344±0.486 | 0.37±0.49 | -0.271 | 0.787 | 0.989282208588957 |
| PMC.L-FPA | (7,37) | 0.399±0.437 | 0.338±0.462 | 0.683 | 0.496 | 0.989282208588957 |
| PMC.L-FPA | (7,38) | 0.178±0.52 | 0.315±0.515 | -1.337 | 0.184 | 0.989282208588957 |
| PMC.L-IFG.L | (7,39) | 0.317±0.477 | 0.388±0.485 | -0.75 | 0.455 | 0.989282208588957 |
| PMC.L-IFG.L | (7,40) | 0.328±0.455 | 0.376±0.481 | -0.514 | 0.608 | 0.989282208588957 |
| PMC.R-PMC.R | (7,41) | 0.264±0.52 | 0.427±0.464 | -1.687 | 0.095 | 0.989282208588957 |
| PMC.R-PMC.R | (7,42) | 0.221±0.538 | 0.333±0.481 | -1.115 | 0.267 | 0.989282208588957 |
| PMC.R-PMC.L | (7,44) | 0.235±0.502 | 0.34±0.457 | -1.115 | 0.267 | 0.989282208588957 |
| PMC.R-PMC.L | (7,45) | 0.205±0.53 | 0.338±0.459 | -1.361 | 0.176 | 0.989282208588957 |
| DLPFC.R-DLPFC.L | (20,22) | 0.601±0.413 | 0.546±0.443 | 0.651 | 0.517 | 0.989282208588957 |
| DLPFC.R-DLPFC.R | (20,26) | 0.473±0.505 | 0.463±0.508 | 0.096 | 0.924 | 0.989282208588957 |
| DLPFC.R-DLPFC.L | (20,27) | 0.55±0.477 | 0.569±0.442 | -0.216 | 0.83 | 0.989282208588957 |
| DLPFC.R-FPA | (20,32) | 0.402±0.552 | 0.45±0.519 | -0.448 | 0.655 | 0.989282208588957 |
| DLPFC.R-IFG.R | (20,35) | 0.486±0.485 | 0.283±0.545 | 1.985 | 0.05 | 0.989282208588957 |
| DLPFC.R-IFG.R | (20,36) | 0.347±0.582 | 0.33±0.489 | 0.158 | 0.874 | 0.989282208588957 |
| DLPFC.R-FPA | (20,37) | 0.464±0.454 | 0.29±0.53 | 1.788 | 0.077 | 0.989282208588957 |
| DLPFC.R-FPA | (20,38) | 0.31±0.527 | 0.204±0.525 | 1.019 | 0.31 | 0.989282208588957 |
| DLPFC.R-IFG.L | (20,39) | 0.376±0.491 | 0.289±0.513 | 0.878 | 0.382 | 0.989282208588957 |
| DLPFC.R-IFG.L | (20,40) | 0.502±0.463 | 0.355±0.545 | 1.473 | 0.144 | 0.989282208588957 |
| DLPFC.R-OFC.R | (20,41) | 0.416±0.539 | 0.408±0.521 | 0.08 | 0.936 | 0.989282208588957 |
| DLPFC.R-OFC.R | (20,42) | 0.247±0.583 | 0.301±0.507 | -0.5 | 0.618 | 0.989282208588957 |
| DLPFC.R-OFC.L | (20,44) | 0.355±0.536 | 0.285±0.583 | 0.635 | 0.527 | 0.989282208588957 |
| DLPFC.R-OFC.L | (20,45) | 0.262±0.542 | 0.253±0.544 | 0.087 | 0.931 | 0.989282208588957 |
| DLPFC.R-DLPFC.L | (22,26) | 0.477±0.509 | 0.506±0.497 | -0.295 | 0.768 | 0.989282208588957 |
| DLPFC.L-DLPFC.L | (22,27) | 0.568±0.4 | 0.623±0.362 | -0.74 | 0.461 | 0.989282208588957 |
| DLPFC.L-FPA | (22,32) | 0.516±0.498 | 0.564±0.429 | -0.526 | 0.6 | 0.989282208588957 |
| DLPFC.L-IFG.R | (22,35) | 0.476±0.474 | 0.248±0.524 | 2.308 | 0.023 | 0.989282208588957 |
| DLPFC.L-IFG.R | (22,36) | 0.356±0.582 | 0.341±0.497 | 0.143 | 0.887 | 0.989282208588957 |
| DLPFC.L-FPA | (22,37) | 0.421±0.524 | 0.319±0.452 | 1.062 | 0.291 | 0.989282208588957 |
| DLPFC.L-FPA | (22,38) | 0.317±0.537 | 0.297±0.513 | 0.19 | 0.849 | 0.989282208588957 |
| DLPFC.L-IFG.L | (22,39) | 0.416±0.409 | 0.337±0.521 | 0.862 | 0.391 | 0.989282208588957 |
| DLPFC.L-IFG.L | (22,40) | 0.531±0.425 | 0.431±0.549 | 1.037 | 0.302 | 0.989282208588957 |
| DLPFC.L-OFC.R | (22,41) | 0.4±0.557 | 0.408±0.514 | -0.072 | 0.943 | 0.989282208588957 |
| DLPFC.L-OFC.R | (22,42) | 0.36±0.537 | 0.424±0.426 | -0.667 | 0.506 | 0.989282208588957 |
| DLPFC.L-OFC.L | (22,44) | 0.404±0.546 | 0.362±0.516 | 0.397 | 0.692 | 0.989282208588957 |
| DLPFC.L-OFC.L | (22,45) | 0.343±0.535 | 0.371±0.43 | -0.29 | 0.772 | 0.989282208588957 |
| DLPFC.R-DLPFC.L | (26,27) | 0.55±0.428 | 0.566±0.429 | -0.191 | 0.849 | 0.989282208588957 |
| DLPFC.R-FPA | (26,32) | 0.504±0.478 | 0.508±0.47 | -0.037 | 0.97 | 0.993233532934132 |
| DLPFC.R-IFG.R | (26,35) | 0.58±0.413 | 0.429±0.468 | 1.731 | 0.087 | 0.989282208588957 |
| DLPFC.R-IFG.R | (26,36) | 0.519±0.486 | 0.393±0.506 | 1.284 | 0.202 | 0.989282208588957 |
| DLPFC.R-FPA | (26,37) | 0.463±0.474 | 0.393±0.486 | 0.745 | 0.458 | 0.989282208588957 |
| DLPFC.R-FPA | (26,38) | 0.302±0.545 | 0.341±0.486 | -0.382 | 0.703 | 0.989282208588957 |
| DLPFC.R-IFG.L | (26,39) | 0.337±0.464 | 0.463±0.407 | -1.464 | 0.146 | 0.989282208588957 |
| DLPFC.R-IFG.L | (26,40) | 0.568±0.401 | 0.423±0.486 | 1.645 | 0.103 | 0.989282208588957 |
| DLPFC.R-OFC.R | (26,41) | 0.407±0.537 | 0.57±0.352 | -1.823 | 0.071 | 0.989282208588957 |
| DLPFC.R-OFC.R | (26,42) | 0.387±0.51 | 0.338±0.467 | 0.51 | 0.611 | 0.989282208588957 |
| DLPFC.R-OFC.L | (26,44) | 0.35±0.598 | 0.474±0.455 | -1.18 | 0.241 | 0.989282208588957 |
| DLPFC.R-OFC.L | (26,45) | 0.294±0.593 | 0.348±0.491 | -0.503 | 0.616 | 0.989282208588957 |
| DLPFC.L-FPA | (27,32) | 0.455±0.478 | 0.479±0.487 | -0.258 | 0.797 | 0.989282208588957 |
| DLPFC.L-IFG.R | (27,35) | 0.53±0.437 | 0.317±0.536 | 2.208 | 0.029 | 0.989282208588957 |
| DLPFC.L-IFG.R | (27,36) | 0.454±0.563 | 0.455±0.438 | -0.012 | 0.991 | 0.994 |
| DLPFC.L-FPA | (27,37) | 0.497±0.431 | 0.43±0.434 | 0.78 | 0.437 | 0.989282208588957 |
| DLPFC.L-FPA | (27,38) | 0.261±0.533 | 0.37±0.497 | -1.076 | 0.285 | 0.989282208588957 |
| DLPFC.L-IFG.L | (27,39) | 0.419±0.418 | 0.343±0.493 | 0.834 | 0.406 | 0.989282208588957 |
| DLPFC.L-IFG.L | (27,40) | 0.558±0.398 | 0.428±0.528 | 1.408 | 0.162 | 0.989282208588957 |
| DLPFC.L-OFC.R | (27,41) | 0.463±0.507 | 0.48±0.484 | -0.165 | 0.869 | 0.989282208588957 |
| DLPFC.L-OFC.R | (27,42) | 0.347±0.537 | 0.396±0.478 | -0.494 | 0.622 | 0.989282208588957 |
| DLPFC.L-OFC.L | (27,44) | 0.406±0.509 | 0.312±0.561 | 0.891 | 0.375 | 0.989282208588957 |
| DLPFC.L-OFC.L | (27,45) | 0.364±0.537 | 0.412±0.445 | -0.488 | 0.627 | 0.989282208588957 |
| FPA-IFG.R | (32,35) | 0.354±0.521 | 0.319±0.505 | 0.345 | 0.731 | 0.989282208588957 |
| FPA-IFG.R | (32,36) | 0.363±0.582 | 0.337±0.532 | 0.237 | 0.813 | 0.989282208588957 |
| FPA-FPA | (32,37) | 0.318±0.537 | 0.31±0.48 | 0.074 | 0.941 | 0.989282208588957 |
| FPA-FPA | (32,38) | 0.147±0.609 | 0.228±0.533 | -0.723 | 0.471 | 0.989282208588957 |
| FPA-IFG.L | (32,39) | 0.451±0.456 | 0.342±0.499 | 1.164 | 0.247 | 0.989282208588957 |
| FPA-IFG.L | (32,40) | 0.413±0.504 | 0.388±0.51 | 0.247 | 0.805 | 0.989282208588957 |
| FPA-OFC.R | (32,41) | 0.345±0.545 | 0.368±0.527 | -0.221 | 0.826 | 0.989282208588957 |
| FPA-OFC.R | (32,42) | 0.618±0.392 | 0.439±0.468 | 2.102 | 0.038 | 0.989282208588957 |
| FPA-OFC.L | (32,44) | 0.238±0.623 | 0.26±0.529 | -0.201 | 0.841 | 0.989282208588957 |
| FPA-OFC.L | (32,45) | 0.348±0.588 | 0.207±0.521 | 1.288 | 0.201 | 0.989282208588957 |
| IFG.R-IFG.R | (35,36) | 0.484±0.513 | 0.43±0.504 | 0.538 | 0.592 | 0.989282208588957 |
| IFG.R-FPA | (35,37) | 0.556±0.442 | 0.39±0.489 | 1.811 | 0.073 | 0.989282208588957 |
| IFG.R-FPA | (35,38) | 0.376±0.528 | 0.361±0.497 | 0.15 | 0.881 | 0.989282208588957 |
| IFG.R-IFG.L | (35,39) | 0.372±0.465 | 0.382±0.425 | -0.117 | 0.907 | 0.989282208588957 |
| IFG.R-IFG.L | (35,40) | 0.56±0.406 | 0.318±0.513 | 2.65 | 0.009 | 0.989282208588957 |
| IFG.R-OFC.R | (35,41) | 0.413±0.553 | 0.334±0.542 | 0.737 | 0.463 | 0.989282208588957 |
| IFG.R-OFC.R | (35,42) | 0.253±0.542 | 0.22±0.489 | 0.323 | 0.748 | 0.989282208588957 |
| IFG.R-OFC.L | (35,44) | 0.308±0.569 | 0.332±0.52 | -0.228 | 0.82 | 0.989282208588957 |
| IFG.R-OFC.L | (35,45) | 0.249±0.595 | 0.235±0.515 | 0.134 | 0.894 | 0.989282208588957 |
| IFG.R-FPA | (36,37) | 0.459±0.485 | 0.286±0.551 | 1.693 | 0.094 | 0.989282208588957 |
| IFG.R-FPA | (36,38) | 0.202±0.624 | 0.241±0.558 | -0.329 | 0.743 | 0.989282208588957 |
| IFG.R-IFG.L | (36,39) | 0.279±0.562 | 0.232±0.513 | 0.436 | 0.664 | 0.989282208588957 |
| IFG.R-IFG.L | (36,40) | 0.43±0.522 | 0.357±0.52 | 0.702 | 0.484 | 0.989282208588957 |
| IFG.R-OFC.R | (36,41) | 0.362±0.594 | 0.282±0.542 | 0.718 | 0.475 | 0.989282208588957 |
| IFG.R-OFC.R | (36,42) | 0.303±0.585 | 0.287±0.524 | 0.141 | 0.888 | 0.989282208588957 |
| IFG.R-OFC.L | (36,44) | 0.374±0.584 | 0.266±0.561 | 0.957 | 0.341 | 0.989282208588957 |
| IFG.R-OFC.L | (36,45) | 0.484±0.544 | 0.471±0.531 | 0.123 | 0.902 | 0.989282208588957 |
| FPA-FPA | (37,38) | 0.455±0.561 | 0.489±0.486 | -0.33 | 0.742 | 0.989282208588957 |
| FPA-IFG.L | (37,39) | 0.462±0.48 | 0.528±0.467 | -0.705 | 0.483 | 0.989282208588957 |
| FPA-IFG.L | (37,40) | 0.548±0.457 | 0.558±0.386 | -0.117 | 0.907 | 0.989282208588957 |
| FPA-OFC.R | (37,41) | 0.385±0.544 | 0.459±0.519 | -0.709 | 0.48 | 0.989282208588957 |
| FPA-OFC.R | (37,42) | 0.279±0.587 | 0.322±0.528 | -0.393 | 0.695 | 0.989282208588957 |
| FPA-OFC.L | (37,44) | 0.379±0.539 | 0.274±0.577 | 0.955 | 0.342 | 0.989282208588957 |
| FPA-OFC.L | (37,45) | 0.424±0.558 | 0.474±0.444 | -0.505 | 0.615 | 0.989282208588957 |
| FPA-IFG.L | (38,39) | 0.466±0.523 | 0.557±0.431 | -0.956 | 0.341 | 0.989282208588957 |
| FPA-IFG.L | (38,40) | 0.49±0.48 | 0.443±0.506 | 0.489 | 0.626 | 0.989282208588957 |
| FPA-OFC.R | (38,41) | 0.376±0.583 | 0.41±0.508 | -0.314 | 0.754 | 0.989282208588957 |
| FPA-OFC.R | (38,42) | 0.295±0.57 | 0.266±0.545 | 0.269 | 0.789 | 0.989282208588957 |
| FPA-OFC.L | (38,44) | 0.289±0.551 | 0.314±0.55 | -0.233 | 0.816 | 0.989282208588957 |
| FPA-OFC.L | (38,45) | 0.28±0.588 | 0.339±0.516 | -0.537 | 0.592 | 0.989282208588957 |
| IFG.L-IFG.L | (39,40) | 0.487±0.433 | 0.478±0.465 | 0.097 | 0.923 | 0.989282208588957 |
| IFG.L-OFC.R | (39,41) | 0.49±0.485 | 0.544±0.449 | -0.578 | 0.565 | 0.989282208588957 |
| IFG.L-OFC.R | (39,42) | 0.392±0.518 | 0.441±0.469 | -0.498 | 0.619 | 0.989282208588957 |
| IFG.L-OFC.L | (39,44) | 0.344±0.509 | 0.349±0.49 | -0.049 | 0.961 | 0.993233532934132 |
| IFG.L-OFC.L | (39,45) | 0.35±0.49 | 0.302±0.479 | 0.503 | 0.616 | 0.989282208588957 |
| IFG.L-OFC.R | (40,41) | 0.508±0.506 | 0.449±0.521 | 0.584 | 0.561 | 0.989282208588957 |
| IFG.L-OFC.R | (40,42) | 0.368±0.57 | 0.5±0.494 | -1.261 | 0.21 | 0.989282208588957 |
| IFG.L-OFC.L | (40,44) | 0.451±0.511 | 0.383±0.548 | 0.649 | 0.518 | 0.989282208588957 |
| IFG.L-OFC.L | (40,45) | 0.41±0.538 | 0.393±0.488 | 0.169 | 0.866 | 0.989282208588957 |
| OFC.R-OFC.R | (41,42) | 0.437±0.52 | 0.473±0.477 | -0.361 | 0.719 | 0.989282208588957 |
| OFC.R-OFC.L | (41,44) | 0.582±0.408 | 0.548±0.426 | 0.422 | 0.674 | 0.989282208588957 |
| OFC.R-OFC.L | (41,45) | 0.382±0.542 | 0.384±0.49 | -0.021 | 0.983 | 0.994 |
| OFC.R-OFC.L | (42,44) | 0.437±0.563 | 0.432±0.519 | 0.049 | 0.961 | 0.993233532934132 |
| OFC.R-OFC.L | (42,45) | 0.336±0.602 | 0.269±0.533 | 0.597 | 0.552 | 0.989282208588957 |
| OFC.L-OFC.L | (44,45) | 0.416±0.559 | 0.379±0.519 | 0.345 | 0.731 | 0.989282208588957 |

The values are the means ± standard deviations.

PMC,premotor area;DLPFC,dorsolateral prefrontal cortex;FPA,frontal pole area; IFG,inferior frontal gyrus; OFC,.orbitofrontal cortex; R, right; L, left

**Table S8. Comparison of functional connectivity strength in 6 weeks post-intervention between the two groups.**

| **Brain regions** | **Channel pairs** | **Experimental** | **Sham** | **t** | ***P*** |  |
| --- | --- | --- | --- | --- | --- | --- |
| PMC.R-PMC.L | (1,3) | 0.445±0.456 | 0.6±0.37 | -1.901 | 0.06 | 0.392837837837838 |
| PMC.R-PMC.R | (1,4) | 0.292±0.418 | 0.278±0.441 | 0.171 | 0.865 | 0.930283018867925 |
| PMC.R-PMC.L | (1,7) | 0.336±0.439 | 0.229±0.437 | 1.248 | 0.215 | 0.503630136986301 |
| PMC.R-DLPFC.R | (1,20) | 0.495±0.361 | 0.364±0.414 | 1.71 | 0.09 | 0.394615384615385 |
| PMC.R-DLPFC.L | (1,22) | 0.453±0.372 | 0.326±0.433 | 1.606 | 0.111 | 0.394615384615385 |
| PMC.R-DLPFC.R | (1,26) | 0.443±0.375 | 0.224±0.497 | 2.516 | 0.013 | 0.202090909090909 |
| PMC.R-DLPFC.L | (1,27) | 0.392±0.424 | 0.329±0.39 | 0.79 | 0.432 | 0.670135135135135 |
| PMC.R-FPA | (1,32) | 0.339±0.47 | 0.165±0.487 | 1.847 | 0.068 | 0.392837837837838 |
| PMC.R-IFG.R | (1,35) | 0.323±0.423 | 0.264±0.39 | 0.735 | 0.464 | 0.688422413793103 |
| PMC.R-IFG.R | (1,36) | 0.383±0.461 | 0.242±0.435 | 1.597 | 0.113 | 0.394615384615385 |
| **PMC.R-FPA** | **(1,37)** | **0.379±0.468** | **0.082±0.47** | **3.203** | **0.002** | 0.057 |
| **PMC.R-FPA** | **(1,38)** | **0.41±0.412** | **0.163±0.444** | **2.921** | **0.004** | 0.076 |
| **PMC.R-IFG.L** | **(1,39)** | **0.229±0.513** | **-0.058±0.459** | **2.994** | **0.003** | 0.064125 |
| PMC.R-IFG.L | (1,40) | 0.386±0.428 | 0.236±0.406 | 1.824 | 0.071 | 0.392837837837838 |
| PMC.R-OFC.R | (1,41) | 0.42±0.411 | 0.223±0.462 | 2.279 | 0.025 | 0.351 |
| PMC.R-OFC.R | (1,42) | 0.27±0.493 | 0.144±0.466 | 1.332 | 0.186 | 0.471471428571429 |
| **PMC.R-OFC.L** | **(1,44)** | **0.445±0.406** | **0.186±0.432** | **3.136** | **0.002** | 0.057 |
| **PMC.R-OFC.L** | **(1,45)** | **0.284±0.483** | **0.09±0.482** | **2.039** | **0.044** | 0.371739130434783 |
| PMC.R-PMC.L | (3,4) | 0.288±0.389 | 0.335±0.444 | -0.58 | 0.564 | 0.76415625 |
| PMC.L-PMC.L | (3,7) | 0.193±0.464 | 0.245±0.494 | -0.551 | 0.583 | 0.764931297709924 |
| PMC.L-DLPFC.R | (3,20) | 0.39±0.464 | 0.379±0.454 | 0.12 | 0.905 | 0.951515337423313 |
| PMC.L-DLPFC.L | (3,22) | 0.392±0.443 | 0.33±0.501 | 0.669 | 0.505 | 0.713677685950413 |
| PMC.L-DLPFC.R | (3,26) | 0.369±0.406 | 0.264±0.496 | 1.174 | 0.243 | 0.536810126582278 |
| PMC.L-DLPFC.L | (3,27) | 0.376±0.416 | 0.424±0.436 | -0.568 | 0.572 | 0.76415625 |
| PMC.L-FPA | (3,32) | 0.278±0.551 | 0.195±0.523 | 0.784 | 0.435 | 0.670135135135135 |
| PMC.L-IFG.R | (3,35) | 0.301±0.407 | 0.259±0.461 | 0.492 | 0.624 | 0.767706293706294 |
| PMC.L-IFG.R | (3,36) | 0.422±0.411 | 0.303±0.479 | 1.353 | 0.179 | 0.471471428571429 |
| PMC.L-FPA | (3,37) | 0.23±0.518 | 0.095±0.528 | 1.311 | 0.193 | 0.471471428571429 |
| PMC.L-FPA | (3,38) | 0.21±0.514 | 0.212±0.477 | -0.016 | 0.987 | 0.992 |
| **PMC.L-IFG.L** | **(3,39)** | **0.111±0.532** | **-0.1±0.489** | **2.096** | **0.039** | 0.351 |
| PMC.L-IFG.L | (3,40) | 0.33±0.479 | 0.2±0.494 | 1.359 | 0.177 | 0.471471428571429 |
| PMC.R-PMC.R | (3,41) | 0.319±0.495 | 0.247±0.508 | 0.729 | 0.467 | 0.688422413793103 |
| PMC.R-PMC.R | (3,42) | 0.221±0.52 | 0.105±0.515 | 1.142 | 0.256 | 0.540204545454545 |
| **PMC.R-PMC.L** | **(3,44)** | **0.365±0.482** | **0.153±0.504** | **2.189** | **0.031** | 0.351 |
| PMC.R-PMC.L | (3,45) | 0.329±0.442 | 0.141±0.52 | 1.98 | 0.05 | 0.371739130434783 |
| PMC.R-PMC.L | (4,7) | 0.173±0.444 | 0.245±0.415 | -0.842 | 0.402 | 0.6365 |
| PMC.R-DLPFC.R | (4,20) | 0.403±0.424 | 0.306±0.425 | 1.164 | 0.247 | 0.536810126582278 |
| PMC.R-DLPFC.L | (4,22) | 0.322±0.414 | 0.271±0.429 | 0.611 | 0.543 | 0.75490243902439 |
| PMC.R-DLPFC.R | (4,26) | 0.311±0.393 | 0.233±0.431 | 0.952 | 0.343 | 0.5985 |
| PMC.R-DLPFC.L | (4,27) | 0.273±0.411 | 0.317±0.416 | -0.538 | 0.592 | 0.766285714285714 |
| PMC.R-FPA | (4,32) | 0.245±0.439 | 0.182±0.418 | 0.752 | 0.454 | 0.687026548672566 |
| PMC.R-IFG.R | (4,35) | 0.213±0.406 | 0.225±0.402 | -0.152 | 0.88 | 0.9405 |
| PMC.R-IFG.R | (4,36) | 0.264±0.458 | 0.238±0.441 | 0.288 | 0.774 | 0.865058823529412 |
| PMC.R-FPA | (4,37) | 0.199±0.457 | 0.106±0.408 | 1.099 | 0.275 | 0.540204545454545 |
| PMC.R-FPA | (4,38) | 0.193±0.41 | 0.237±0.348 | -0.579 | 0.564 | 0.76415625 |
| PMC.R-IFG.L | (4,39) | 0.067±0.459 | 0.027±0.367 | 0.495 | 0.622 | 0.767706293706294 |
| PMC.R-IFG.L | (4,40) | 0.278±0.429 | 0.149±0.451 | 1.476 | 0.143 | 0.436344827586207 |
| PMC.R-OFC.R | (4,41) | 0.257±0.386 | 0.199±0.423 | 0.717 | 0.475 | 0.694230769230769 |
| PMC.R-OFC.R | (4,42) | 0.162±0.454 | 0.131±0.395 | 0.37 | 0.712 | 0.822648648648649 |
| PMC.R-OFC.L | (4,44) | 0.27±0.424 | 0.121±0.436 | 1.749 | 0.083 | 0.392837837837838 |
| PMC.R-OFC.L | (4,45) | 0.149±0.439 | 0.13±0.464 | 0.209 | 0.835 | 0.921193548387097 |
| PMC.L-DLPFC.R | (7,20) | 0.433±0.448 | 0.374±0.438 | 0.673 | 0.503 | 0.713677685950413 |
| PMC.L-DLPFC.L | (7,22) | 0.341±0.495 | 0.301±0.455 | 0.425 | 0.672 | 0.782877551020408 |
| PMC.L-DLPFC.R | (7,26) | 0.345±0.475 | 0.484±0.365 | -1.669 | 0.098 | 0.394615384615385 |
| PMC.L-DLPFC.L | (7,27) | 0.312±0.47 | 0.345±0.487 | -0.349 | 0.728 | 0.82992 |
| PMC.L-FPA | (7,32) | 0.289±0.544 | 0.364±0.431 | -0.772 | 0.442 | 0.674839285714286 |
| PMC.L-IFG.R | (7,35) | 0.3±0.444 | 0.363±0.451 | -0.708 | 0.48 | 0.695593220338983 |
| PMC.L-IFG.R | (7,36) | 0.33±0.521 | 0.284±0.466 | 0.467 | 0.642 | 0.767706293706294 |
| PMC.L-FPA | (7,37) | 0.345±0.462 | 0.248±0.436 | 1.095 | 0.276 | 0.540204545454545 |
| PMC.L-FPA | (7,38) | 0.382±0.409 | 0.343±0.437 | 0.466 | 0.642 | 0.767706293706294 |
| PMC.L-IFG.L | (7,39) | 0.303±0.458 | 0.184±0.444 | 1.335 | 0.185 | 0.471471428571429 |
| PMC.L-IFG.L | (7,40) | 0.358±0.474 | 0.348±0.468 | 0.107 | 0.915 | 0.954054878048781 |
| PMC.R-PMC.R | (7,41) | 0.429±0.401 | 0.207±0.488 | 2.528 | 0.013 | 0.202090909090909 |
| PMC.R-PMC.R | (7,42) | 0.333±0.456 | 0.254±0.474 | 0.856 | 0.394 | 0.629663551401869 |
| **PMC.R-PMC.L** | **(7,44)** | **0.404±0.489** | **0.189±0.542** | **2.113** | **0.037** | 0.351 |
| PMC.R-PMC.L | (7,45) | 0.257±0.506 | 0.161±0.491 | 0.979 | 0.33 | 0.581752577319588 |
| DLPFC.R-DLPFC.L | (20,22) | 0.667±0.377 | 0.654±0.356 | 0.175 | 0.861 | 0.930283018867925 |
| **DLPFC.R-DLPFC.R** | **(20,26)** | **0.648±0.323** | **0.37±0.495** | **3.368** | **0.001** | 0.057 |
| DLPFC.R-DLPFC.L | (20,27) | 0.616±0.351 | 0.487±0.436 | 1.653 | 0.101 | 0.394615384615385 |
| DLPFC.R-FPA | (20,32) | 0.444±0.516 | 0.333±0.489 | 1.125 | 0.263 | 0.540204545454545 |
| DLPFC.R-IFG.R | (20,35) | 0.437±0.439 | 0.342±0.447 | 1.091 | 0.278 | 0.540204545454545 |
| DLPFC.R-IFG.R | (20,36) | 0.521±0.499 | 0.411±0.459 | 1.163 | 0.248 | 0.536810126582278 |
| DLPFC.R-FPA | (20,37) | 0.42±0.496 | 0.32±0.446 | 1.08 | 0.283 | 0.543741573033708 |
| DLPFC.R-FPA | (20,38) | 0.477±0.428 | 0.36±0.422 | 1.403 | 0.164 | 0.4674 |
| DLPFC.R-IFG.L | (20,39) | 0.337±0.531 | 0.159±0.489 | 1.763 | 0.081 | 0.392837837837838 |
| DLPFC.R-IFG.L | (20,40) | 0.505±0.413 | 0.34±0.481 | 1.866 | 0.065 | 0.392837837837838 |
| DLPFC.R-OFC.R | (20,41) | 0.518±0.409 | 0.363±0.468 | 1.787 | 0.077 | 0.392837837837838 |
| DLPFC.R-OFC.R | (20,42) | 0.379±0.495 | 0.33±0.532 | 0.479 | 0.633 | 0.767706293706294 |
| DLPFC.R-OFC.L | (20,44) | 0.555±0.431 | 0.4±0.46 | 1.769 | 0.08 | 0.392837837837838 |
| DLPFC.R-OFC.L | (20,45) | 0.391±0.495 | 0.289±0.512 | 1.028 | 0.306 | 0.568760869565217 |
| **DLPFC.R-DLPFC.L** | **(22,26)** | **0.649±0.315** | **0.365±0.479** | **3.545** | **0.001** | 0.057 |
| DLPFC.L-DLPFC.L | (22,27) | 0.655±0.322 | 0.536±0.434 | 1.576 | 0.118 | 0.394615384615385 |
| DLPFC.L-FPA | (22,32) | 0.598±0.404 | 0.456±0.465 | 1.652 | 0.102 | 0.394615384615385 |
| DLPFC.L-IFG.R | (22,35) | 0.429±0.397 | 0.353±0.436 | 0.926 | 0.357 | 0.604425742574257 |
| DLPFC.L-IFG.R | (22,36) | 0.541±0.426 | 0.463±0.45 | 0.902 | 0.369 | 0.615932038834951 |
| DLPFC.L-FPA | (22,37) | 0.435±0.459 | 0.27±0.497 | 1.746 | 0.084 | 0.392837837837838 |
| DLPFC.L-FPA | (22,38) | 0.408±0.433 | 0.318±0.502 | 0.98 | 0.33 | 0.581752577319588 |
| DLPFC.L-IFG.L | (22,39) | 0.368±0.476 | 0.21±0.501 | 1.645 | 0.103 | 0.394615384615385 |
| DLPFC.L-IFG.L | (22,40) | 0.486±0.436 | 0.376±0.474 | 1.227 | 0.223 | 0.50844 |
| DLPFC.L-OFC.R | (22,41) | 0.492±0.442 | 0.403±0.466 | 0.992 | 0.324 | 0.581752577319588 |
| DLPFC.L-OFC.R | (22,42) | 0.426±0.441 | 0.419±0.46 | 0.083 | 0.934 | 0.962132530120482 |
| DLPFC.L-OFC.L | (22,44) | 0.513±0.443 | 0.431±0.444 | 0.935 | 0.352 | 0.60192 |
| DLPFC.L-OFC.L | (22,45) | 0.483±0.407 | 0.319±0.526 | 1.769 | 0.08 | 0.392837837837838 |
| DLPFC.R-DLPFC.L | (26,27) | 0.511±0.311 | 0.439±0.372 | 1.063 | 0.29 | 0.551 |
| DLPFC.R-FPA | (26,32) | 0.327±0.474 | 0.434±0.379 | -1.274 | 0.206 | 0.48925 |
| DLPFC.R-IFG.R | (26,35) | 0.421±0.377 | 0.368±0.35 | 0.731 | 0.466 | 0.688422413793103 |
| DLPFC.R-IFG.R | (26,36) | 0.416±0.438 | 0.325±0.463 | 1.033 | 0.304 | 0.568760869565217 |
| DLPFC.R-FPA | (26,37) | 0.335±0.478 | 0.284±0.434 | 0.567 | 0.572 | 0.76415625 |
| DLPFC.R-FPA | (26,38) | 0.372±0.403 | 0.234±0.441 | 1.655 | 0.101 | 0.394615384615385 |
| DLPFC.R-IFG.L | (26,39) | 0.295±0.457 | 0.154±0.439 | 1.587 | 0.116 | 0.394615384615385 |
| DLPFC.R-IFG.L | (26,40) | 0.414±0.415 | 0.299±0.445 | 1.358 | 0.178 | 0.471471428571429 |
| DLPFC.R-OFC.R | (26,41) | 0.443±0.398 | 0.274±0.41 | 2.121 | 0.036 | 0.351 |
| DLPFC.R-OFC.R | (26,42) | 0.286±0.463 | 0.313±0.443 | -0.307 | 0.759 | 0.853875 |
| DLPFC.R-OFC.L | (26,44) | 0.446±0.421 | 0.32±0.463 | 1.445 | 0.152 | 0.440542372881356 |
| **DLPFC.R-OFC.L** | **(26,45)** | **0.355±0.431** | **0.174±0.485** | **1.997** | **0.049** | 0.371739130434783 |
| DLPFC.L-FPA | (27,32) | 0.461±0.43 | 0.343±0.48 | 1.312 | 0.192 | 0.471471428571429 |
| DLPFC.L-IFG.R | (27,35) | 0.426±0.36 | 0.478±0.389 | -0.7 | 0.485 | 0.696932773109244 |
| DLPFC.L-IFG.R | (27,36) | 0.583±0.399 | 0.572±0.378 | 0.141 | 0.888 | 0.943155279503106 |
| DLPFC.L-FPA | (27,37) | 0.482±0.425 | 0.366±0.47 | 1.316 | 0.191 | 0.471471428571429 |
| DLPFC.L-FPA | (27,38) | 0.41±0.453 | 0.454±0.431 | -0.514 | 0.608 | 0.767706293706294 |
| DLPFC.L-IFG.L | (27,39) | 0.351±0.483 | 0.176±0.479 | 1.841 | 0.069 | 0.392837837837838 |
| DLPFC.L-IFG.L | (27,40) | 0.483±0.448 | 0.434±0.472 | 0.532 | 0.596 | 0.766285714285714 |
| DLPFC.L-OFC.R | (27,41) | 0.474±0.476 | 0.478±0.427 | -0.04 | 0.968 | 0.991185628742515 |
| DLPFC.L-OFC.R | (27,42) | 0.41±0.46 | 0.27±0.511 | 1.458 | 0.148 | 0.436344827586207 |
| DLPFC.L-OFC.L | (27,44) | 0.48±0.437 | 0.469±0.462 | 0.117 | 0.907 | 0.951515337423313 |
| DLPFC.L-OFC.L | (27,45) | 0.513±0.366 | 0.38±0.485 | 1.57 | 0.12 | 0.394615384615385 |
| FPA-IFG.R | (32,35) | 0.28±0.499 | 0.247±0.5 | 0.334 | 0.739 | 0.836880794701987 |
| FPA-IFG.R | (32,36) | 0.429±0.537 | 0.402±0.519 | 0.259 | 0.796 | 0.88387012987013 |
| FPA-FPA | (32,37) | 0.398±0.512 | 0.35±0.468 | 0.49 | 0.625 | 0.767706293706294 |
| FPA-FPA | (32,38) | 0.287±0.567 | 0.267±0.507 | 0.188 | 0.851 | 0.930283018867925 |
| FPA-IFG.L | (32,39) | 0.352±0.534 | 0.302±0.477 | 0.504 | 0.616 | 0.767706293706294 |
| FPA-IFG.L | (32,40) | 0.362±0.587 | 0.351±0.521 | 0.1 | 0.921 | 0.954490909090909 |
| FPA-OFC.R | (32,41) | 0.404±0.578 | 0.355±0.533 | 0.452 | 0.652 | 0.77425 |
| FPA-OFC.R | (32,42) | 0.556±0.423 | 0.448±0.465 | 1.228 | 0.222 | 0.50844 |
| FPA-OFC.L | (32,44) | 0.417±0.61 | 0.267±0.496 | 1.372 | 0.173 | 0.471471428571429 |
| FPA-OFC.L | (32,45) | 0.414±0.555 | 0.254±0.502 | 1.531 | 0.129 | 0.416207547169811 |
| IFG.R-IFG.R | (35,36) | 0.456±0.464 | 0.493±0.428 | -0.423 | 0.673 | 0.782877551020408 |
| IFG.R-FPA | (35,37) | 0.383±0.495 | 0.298±0.492 | 0.872 | 0.385 | 0.621084905660377 |
| IFG.R-FPA | (35,38) | 0.426±0.506 | 0.328±0.47 | 1.013 | 0.313 | 0.569393617021277 |
| **IFG.R-IFG.L** | **(35,39)** | **0.429±0.47** | **0.151±0.466** | **3.012** | **0.003** | 0.064125 |
| IFG.R-IFG.L | (35,40) | 0.396±0.463 | 0.346±0.46 | 0.548 | 0.585 | 0.764931297709924 |
| IFG.R-OFC.R | (35,41) | 0.417±0.47 | 0.306±0.485 | 1.184 | 0.239 | 0.536810126582278 |
| IFG.R-OFC.R | (35,42) | 0.216±0.501 | 0.172±0.505 | 0.445 | 0.657 | 0.774806896551724 |
| IFG.R-OFC.L | (35,44) | 0.423±0.462 | 0.379±0.454 | 0.494 | 0.623 | 0.767706293706294 |
| **IFG.R-OFC.L** | **(35,45)** | **0.395±0.433** | **0.193±0.519** | **2.14** | **0.035** | 0.351 |
| IFG.R-FPA | (36,37) | 0.443±0.539 | 0.355±0.553 | 0.814 | 0.417 | 0.654192660550459 |
| IFG.R-FPA | (36,38) | 0.399±0.574 | 0.381±0.504 | 0.174 | 0.862 | 0.930283018867925 |
| IFG.R-IFG.L | (36,39) | 0.283±0.608 | 0.18±0.491 | 0.946 | 0.347 | 0.599363636363636 |
| IFG.R-IFG.L | (36,40) | 0.518±0.521 | 0.52±0.405 | -0.024 | 0.981 | 0.992 |
| IFG.R-OFC.R | (36,41) | 0.494±0.517 | 0.497±0.392 | -0.027 | 0.979 | 0.992 |
| IFG.R-OFC.R | (36,42) | 0.375±0.584 | 0.249±0.527 | 1.142 | 0.256 | 0.540204545454545 |
| IFG.R-OFC.L | (36,44) | 0.6±0.472 | 0.558±0.428 | 0.467 | 0.642 | 0.767706293706294 |
| IFG.R-OFC.L | (36,45) | 0.633±0.383 | 0.539±0.472 | 1.11 | 0.269 | 0.540204545454545 |
| **FPA-FPA** | **(37,38)** | **0.697±0.337** | **0.423±0.523** | **3.151** | **0.002** | 0.057 |
| FPA-IFG.L | (37,39) | 0.487±0.441 | 0.369±0.463 | 1.33 | 0.186 | 0.471471428571429 |
| FPA-IFG.L | (37,40) | 0.523±0.464 | 0.365±0.494 | 1.678 | 0.096 | 0.394615384615385 |
| FPA-OFC.R | (37,41) | 0.528±0.464 | 0.329±0.542 | 2.008 | 0.047 | 0.371739130434783 |
| FPA-OFC.R | (37,42) | 0.435±0.475 | 0.284±0.497 | 1.575 | 0.118 | 0.394615384615385 |
| FPA-OFC.L | (37,44) | 0.453±0.549 | 0.345±0.534 | 1.015 | 0.313 | 0.569393617021277 |
| FPA-OFC.L | (37,45) | 0.512±0.539 | 0.341±0.536 | 1.619 | 0.109 | 0.394615384615385 |
| FPA-IFG.L | (38,39) | 0.554±0.507 | 0.343±0.515 | 2.099 | 0.038 | 0.351 |
| FPA-IFG.L | (38,40) | 0.503±0.497 | 0.45±0.48 | 0.547 | 0.586 | 0.764931297709924 |
| FPA-OFC.R | (38,41) | 0.592±0.394 | 0.41±0.471 | 2.13 | 0.036 | 0.351 |
| FPA-OFC.R | (38,42) | 0.342±0.555 | 0.243±0.561 | 0.898 | 0.371 | 0.615932038834951 |
| FPA-OFC.L | (38,44) | 0.477±0.476 | 0.374±0.482 | 1.098 | 0.275 | 0.540204545454545 |
| FPA-OFC.L | (38,45) | 0.417±0.566 | 0.322±0.527 | 0.879 | 0.382 | 0.621084905660377 |
| IFG.L-IFG.L | (39,40) | 0.387±0.539 | 0.226±0.496 | 1.581 | 0.117 | 0.394615384615385 |
| IFG.L-OFC.R | (39,41) | 0.519±0.456 | 0.385±0.45 | 1.505 | 0.136 | 0.430666666666667 |
| IFG.L-OFC.R | (39,42) | 0.479±0.437 | 0.345±0.499 | 1.457 | 0.148 | 0.436344827586207 |
| IFG.L-OFC.L | (39,44) | 0.417±0.532 | 0.292±0.466 | 1.275 | 0.205 | 0.48925 |
| **IFG.L-OFC.L** | **(39,45)** | **0.508±0.452** | **0.197±0.518** | **3.243** | **0.002** | 0.057 |
| IFG.L-OFC.R | (40,41) | 0.592±0.441 | 0.49±0.48 | 1.127 | 0.262 | 0.540204545454545 |
| IFG.L-OFC.R | (40,42) | 0.462±0.524 | 0.401±0.529 | 0.593 | 0.555 | 0.76415625 |
| IFG.L-OFC.L | (40,44) | 0.596±0.366 | 0.438±0.505 | 1.819 | 0.072 | 0.392837837837838 |
| IFG.L-OFC.L | (40,45) | 0.489±0.513 | 0.337±0.542 | 1.461 | 0.147 | 0.436344827586207 |
| OFC.R-OFC.R | (41,42) | 0.55±0.444 | 0.492±0.475 | 0.645 | 0.521 | 0.730254098360656 |
| OFC.R-OFC.L | (41,44) | 0.661±0.456 | 0.662±0.31 | -0.01 | 0.992 | 0.992 |
| OFC.R-OFC.L | (41,45) | 0.425±0.572 | 0.513±0.434 | -0.881 | 0.38 | 0.621084905660377 |
| OFC.R-OFC.L | (42,44) | 0.536±0.518 | 0.353±0.553 | 1.739 | 0.085 | 0.392837837837838 |
| OFC.R-OFC.L | (42,45) | 0.501±0.51 | 0.307±0.549 | 1.851 | 0.067 | 0.392837837837838 |
| OFC.L-OFC.L | (44,45) | 0.582±0.493 | 0.612±0.368 | -0.351 | 0.726 | 0.82992 |

The values are the means ± standard deviations.

PMC,premotor area;DLPFC,dorsolateral prefrontal cortex;FPA,frontal pole area; IFG,inferior frontal gyrus; OFC,.orbitofrontal cortex; R, right; L, left

**Table S9. Comparison of changes in functional connectivity strength(△) between the two groups.**

| **Brain regions** | **Channel pairs** | **Experimental** | **Sham** | **t** | ***P*** | **FDR** |
| --- | --- | --- | --- | --- | --- | --- |
| PMC.R-PMC.L | (1,3) | -0.146±0.564 | 0.039±0.497 | -1.761 | 0.081 | 0.565615384615385 |
| PMC.R-PMC.R | (1,4) | -0.007±0.525 | -0.059±0.603 | 0.466 | 0.642 | 0.875727272727273 |
| PMC.R-PMC.L | (1,7) | -0.063±0.562 | -0.171±0.622 | 0.922 | 0.359 | 0.836513513513514 |
| PMC.R-DLPFC.R | (1,20) | 0.112±0.48 | 0.058±0.645 | 0.479 | 0.633 | 0.875727272727273 |
| PMC.R-DLPFC.L | (1,22) | 0.129±0.452 | -0.055±0.586 | 1.787 | 0.077 | 0.565615384615385 |
| PMC.R-DLPFC.R | (1,26) | 0.071±0.481 | -0.17±0.666 | 2.105 | 0.038 | 0.361 |
| PMC.R-DLPFC.L | (1,27) | -0.022±0.479 | -0.055±0.618 | 0.307 | 0.759 | 0.916415492957746 |
| PMC.R-FPA | (1,32) | 0.037±0.636 | -0.107±0.539 | 1.242 | 0.217 | 0.639775862068965 |
| PMC.R-IFG.R | (1,35) | -0.07±0.525 | -0.041±0.59 | -0.261 | 0.794 | 0.919268456375839 |
| PMC.R-IFG.R | (1,36) | 0.113±0.643 | -0.071±0.687 | 1.399 | 0.165 | 0.619471698113208 |
| PMC.R-FPA | (1,37) | -0.014±0.563 | -0.241±0.616 | 1.955 | 0.053 | 0.45315 |
| **PMC.R-FPA** | **(1,38)** | **0.297±0.554** | **-0.122±0.624** | **3.602** | **0.000492** | **0.042066** |
| PMC.R-IFG.L | (1,39) | **0.024±0.633** | **-0.341±0.635** | 2.922 | 0.004 | 0.114 |
| PMC.R-IFG.L | (1,40) | 0.082±0.528 | -0.083±0.609 | 1.466 | 0.146 | 0.619471698113208 |
| **PMC.R-OFC.R** | **(1,41)** | **0.145±0.56** | **-0.094±0.596** | **2.102** | **0.038** | 0.361 |
| PMC.R-OFC.R | (1,42) | 0.08±0.582 | -0.023±0.523 | 0.952 | 0.343 | 0.826098591549296 |
| **PMC.R-OFC.L** | **(1,44)** | **0.269±0.576** | **-0.121±0.61** | **3.334** | **0.001** | **0.04275** |
| PMC.R-OFC.L | (1,45) | 0.094±0.653 | -0.208±0.645 | 2.357 | 0.02 | 0.285 |
| PMC.R-PMC.L | (3,4) | -0.062±0.523 | -0.03±0.592 | -0.287 | 0.775 | 0.919268456375839 |
| PMC.L-PMC.L | (3,7) | -0.23±0.542 | -0.075±0.687 | -1.271 | 0.207 | 0.639 |
| PMC.L-DLPFC.R | (3,20) | -0.055±0.677 | 0.01±0.697 | -0.485 | 0.629 | 0.875727272727273 |
| PMC.L-DLPFC.L | (3,22) | -0.005±0.621 | -0.013±0.669 | 0.058 | 0.954 | 0.985825301204819 |
| PMC.L-DLPFC.R | (3,26) | -0.111±0.556 | -0.13±0.71 | 0.144 | 0.886 | 0.951652173913043 |
| PMC.L-DLPFC.L | (3,27) | -0.092±0.512 | 0.106±0.683 | -1.665 | 0.099 | 0.610714285714286 |
| PMC.L-FPA | (3,32) | -0.073±0.75 | -0.152±0.696 | 0.554 | 0.581 | 0.875727272727273 |
| PMC.L-IFG.R | (3,35) | -0.152±0.596 | 0.031±0.674 | -1.459 | 0.148 | 0.619471698113208 |
| PMC.L-IFG.R | (3,36) | 0.073±0.544 | 0.031±0.776 | 0.318 | 0.751 | 0.916415492957746 |
| PMC.L-FPA | (3,37) | -0.154±0.71 | -0.13±0.676 | -0.181 | 0.857 | 0.951603896103896 |
| PMC.L-FPA | (3,38) | 0.088±0.749 | 0.108±0.715 | -0.134 | 0.894 | 0.951652173913043 |
| PMC.L-IFG.L | (3,39) | -0.131±0.642 | -0.261±0.703 | 0.98 | 0.329 | 0.815347826086957 |
| PMC.L-IFG.L | (3,40) | -0.069±0.61 | -0.023±0.757 | -0.343 | 0.732 | 0.90704347826087 |
| PMC.R-PMC.R | (3,41) | 0.062±0.701 | -0.117±0.658 | 1.343 | 0.182 | 0.619471698113208 |
| PMC.R-PMC.R | (3,42) | 0.003±0.686 | -0.048±0.63 | 0.391 | 0.697 | 0.882866666666667 |
| **PMC.R-PMC.L** | **(3,44)** | **0.182±0.605** | **-0.131±0.631** | **2.571** | **0.012** | 0.264272727272727 |
| PMC.R-PMC.L | (3,45) | 0.096±0.653 | -0.081±0.774 | 1.257 | 0.212 | 0.639 |
| PMC.R-PMC.L | (4,7) | -0.169±0.56 | -0.053±0.563 | -1.054 | 0.295 | 0.752910447761194 |
| PMC.R-DLPFC.R | (4,20) | 0.131±0.615 | 0.038±0.654 | 0.74 | 0.461 | 0.875727272727273 |
| PMC.R-DLPFC.L | (4,22) | 0.059±0.613 | -0.031±0.638 | 0.734 | 0.465 | 0.875727272727273 |
| PMC.R-DLPFC.R | (4,26) | -0.037±0.501 | -0.104±0.625 | 0.599 | 0.551 | 0.875727272727273 |
| PMC.R-DLPFC.L | (4,27) | -0.04±0.497 | 0.008±0.64 | -0.427 | 0.67 | 0.875727272727273 |
| PMC.R-FPA | (4,32) | 0.001±0.584 | -0.124±0.57 | 1.102 | 0.273 | 0.733984615384615 |
| PMC.R-IFG.R | (4,35) | -0.057±0.522 | -0.079±0.537 | 0.21 | 0.834 | 0.93825 |
| PMC.R-IFG.R | (4,36) | -0.034±0.642 | 0.021±0.65 | -0.434 | 0.665 | 0.875727272727273 |
| PMC.R-FPA | (4,37) | -0.11±0.515 | -0.081±0.614 | -0.258 | 0.797 | 0.919268456375839 |
| PMC.R-FPA | (4,38) | -0.012±0.606 | 0.146±0.669 | -1.257 | 0.212 | 0.639 |
| PMC.R-IFG.L | (4,39) | -0.085±0.462 | -0.122±0.58 | 0.352 | 0.726 | 0.906175182481752 |
| PMC.R-IFG.L | (4,40) | 0.031±0.581 | -0.049±0.675 | 0.649 | 0.518 | 0.875727272727273 |
| PMC.R-OFC.R | (4,41) | -0.016±0.527 | -0.061±0.691 | 0.371 | 0.712 | 0.895235294117647 |
| PMC.R-OFC.R | (4,42) | -0.089±0.52 | 0.026±0.606 | -1.027 | 0.307 | 0.772014705882353 |
| PMC.R-OFC.L | (4,44) | 0.023±0.56 | -0.078±0.669 | 0.83 | 0.408 | 0.875727272727273 |
| PMC.R-OFC.L | (4,45) | -0.037±0.592 | -0.016±0.678 | -0.163 | 0.871 | 0.951652173913043 |
| PMC.L-DLPFC.R | (7,20) | 0.063±0.601 | -0.024±0.62 | 0.727 | 0.469 | 0.875727272727273 |
| PMC.L-DLPFC.L | (7,22) | 0.018±0.613 | -0.11±0.597 | 1.072 | 0.286 | 0.741 |
| PMC.L-DLPFC.R | (7,26) | -0.038±0.656 | 0.055±0.527 | -0.799 | 0.426 | 0.875727272727273 |
| PMC.L-DLPFC.L | (7,27) | -0.076±0.633 | -0.144±0.666 | 0.534 | 0.595 | 0.875727272727273 |
| PMC.L-FPA | (7,32) | -0.029±0.681 | 0.032±0.673 | -0.451 | 0.653 | 0.875727272727273 |
| PMC.L-IFG.R | (7,35) | -0.029±0.598 | 0.083±0.639 | -0.915 | 0.362 | 0.836513513513514 |
| PMC.L-IFG.R | (7,36) | -0.017±0.619 | -0.086±0.675 | 0.541 | 0.589 | 0.875727272727273 |
| PMC.L-FPA | (7,37) | -0.06±0.677 | -0.09±0.621 | 0.235 | 0.815 | 0.9291 |
| PMC.L-FPA | (7,38) | 0.208±0.589 | 0.029±0.687 | 1.422 | 0.158 | 0.619471698113208 |
| PMC.L-IFG.L | (7,39) | -0.023±0.63 | -0.204±0.627 | 1.464 | 0.146 | 0.619471698113208 |
| PMC.L-IFG.L | (7,40) | 0.025±0.559 | -0.028±0.599 | 0.468 | 0.641 | 0.875727272727273 |
| **PMC.R-PMC.R** | **(7,41)** | **0.165±0.608** | **-0.221±0.596** | **3.252** | **0.002** | 0.0684 |
| PMC.R-PMC.R | (7,42) | 0.106±0.681 | -0.079±0.689 | 1.37 | 0.174 | 0.619471698113208 |
| **PMC.R-PMC.L** | **(7,44)** | **0.179±0.653** | **-0.151±0.667** | **2.538** | **0.013** | 0.264272727272727 |
| PMC.R-PMC.L | (7,45) | 0.051±0.669 | -0.176±0.665 | 1.732 | 0.086 | 0.565615384615385 |
| DLPFC.R-DLPFC.L | (20,22) | 0.065±0.497 | 0.109±0.544 | -0.421 | 0.674 | 0.875727272727273 |
| **DLPFC.R-DLPFC.R** | **(20,26)** | **0.182±0.561** | **-0.093±0.717** | **2.165** | **0.033** | 0.3526875 |
| DLPFC.R-DLPFC.L | (20,27) | 0.065±0.572 | -0.082±0.663 | 1.205 | 0.231 | 0.65835 |
| DLPFC.R-FPA | (20,32) | 0.043±0.717 | -0.117±0.744 | 1.111 | 0.269 | 0.733984615384615 |
| DLPFC.R-IFG.R | (20,35) | -0.046±0.596 | 0.059±0.732 | -0.795 | 0.428 | 0.875727272727273 |
| DLPFC.R-IFG.R | (20,36) | 0.176±0.709 | 0.08±0.609 | 0.736 | 0.464 | 0.875727272727273 |
| DLPFC.R-FPA | (20,37) | -0.049±0.647 | 0.03±0.661 | -0.609 | 0.544 | 0.875727272727273 |
| DLPFC.R-FPA | (20,38) | 0.171±0.59 | 0.155±0.638 | 0.131 | 0.896 | 0.951652173913043 |
| DLPFC.R-IFG.L | (20,39) | -0.043±0.699 | -0.129±0.677 | 0.635 | 0.527 | 0.875727272727273 |
| DLPFC.R-IFG.L | (20,40) | 0±0.621 | -0.015±0.716 | 0.117 | 0.907 | 0.957388888888889 |
| DLPFC.R-OFC.R | (20,41) | 0.101±0.576 | -0.045±0.638 | 1.216 | 0.227 | 0.657915254237288 |
| DLPFC.R-OFC.R | (20,42) | 0.131±0.698 | 0.03±0.734 | 0.72 | 0.473 | 0.875727272727273 |
| DLPFC.R-OFC.L | (20,44) | 0.203±0.67 | 0.115±0.709 | 0.652 | 0.516 | 0.875727272727273 |
| DLPFC.R-OFC.L | (20,45) | 0.129±0.658 | 0.037±0.752 | 0.661 | 0.51 | 0.875727272727273 |
| **DLPFC.R-DLPFC.L** | **(22,26)** | **0.175±0.538** | **-0.141±0.735** | **2.487** | **0.015** | 0.264272727272727 |
| DLPFC.L-DLPFC.L | (22,27) | 0.084±0.492 | -0.087±0.593 | 1.589 | 0.115 | 0.619471698113208 |
| DLPFC.L-FPA | (22,32) | 0.086±0.579 | -0.108±0.603 | 1.659 | 0.1 | 0.610714285714286 |
| DLPFC.L-IFG.R | (22,35) | -0.049±0.574 | 0.105±0.667 | -1.252 | 0.213 | 0.639 |
| DLPFC.L-IFG.R | (22,36) | 0.188±0.662 | 0.123±0.61 | 0.519 | 0.605 | 0.875727272727273 |
| DLPFC.L-FPA | (22,37) | 0.009±0.705 | -0.048±0.647 | 0.43 | 0.668 | 0.875727272727273 |
| DLPFC.L-FPA | (22,38) | 0.091±0.62 | 0.02±0.748 | 0.52 | 0.604 | 0.875727272727273 |
| DLPFC.L-IFG.L | (22,39) | -0.049±0.609 | -0.127±0.752 | 0.579 | 0.564 | 0.875727272727273 |
| DLPFC.L-IFG.L | (22,40) | -0.05±0.562 | -0.055±0.692 | 0.043 | 0.966 | 0.989137724550898 |
| DLPFC.L-OFC.R | (22,41) | 0.089±0.65 | -0.004±0.677 | 0.71 | 0.48 | 0.875727272727273 |
| DLPFC.L-OFC.R | (22,42) | 0.068±0.543 | -0.005±0.634 | 0.626 | 0.533 | 0.875727272727273 |
| DLPFC.L-OFC.L | (22,44) | 0.104±0.572 | 0.069±0.702 | 0.28 | 0.78 | 0.919268456375839 |
| DLPFC.L-OFC.L | (22,45) | 0.136±0.642 | -0.052±0.666 | 1.46 | 0.147 | 0.619471698113208 |
| DLPFC.R-DLPFC.L | (26,27) | 0.121±0.424 | -0.05±0.647 | 1.58 | 0.117 | 0.619471698113208 |
| DLPFC.R-FPA | (26,32) | -0.047±0.594 | -0.029±0.597 | -0.151 | 0.88 | 0.951652173913043 |
| DLPFC.R-IFG.R | (26,35) | -0.124±0.574 | 0.032±0.627 | -1.314 | 0.192 | 0.619471698113208 |
| DLPFC.R-IFG.R | (26,36) | 0.03±0.694 | -0.026±0.776 | 0.39 | 0.697 | 0.882866666666667 |
| DLPFC.R-FPA | (26,37) | 0±0.627 | -0.026±0.712 | 0.195 | 0.846 | 0.945529411764706 |
| DLPFC.R-FPA | (26,38) | 0.159±0.689 | 0.099±0.658 | 0.452 | 0.652 | 0.875727272727273 |
| DLPFC.R-IFG.L | (26,39) | 0.049±0.511 | -0.188±0.55 | 2.269 | 0.025 | 0.305357142857143 |
| DLPFC.R-IFG.L | (26,40) | -0.035±0.557 | -0.071±0.62 | 0.305 | 0.761 | 0.916415492957746 |
| **DLPFC.R-OFC.R** | **(26,41)** | **0.203±0.665** | **-0.264±0.593** | **3.769** | **0.000276** | **0.042066** |
| DLPFC.R-OFC.R | (26,42) | 0.04±0.596 | 0.007±0.688 | 0.253 | 0.801 | 0.919268456375839 |
| **DLPFC.R-OFC.L** | **(26,44)** | **0.223±0.615** | **-0.19±0.606** | **3.43** | **0.001** | **0.04275** |
| DLPFC.R-OFC.L | (26,45) | 0.163±0.716 | -0.113±0.758 | 1.905 | 0.06 | 0.474136363636364 |
| DLPFC.L-FPA | (27,32) | 0.006±0.592 | -0.136±0.725 | 1.089 | 0.279 | 0.733984615384615 |
| **DLPFC.L-IFG.R** | **(27,35)** | **-0.1±0.455** | **0.161±0.666** | **-2.316** | **0.023** | 0.302538461538462 |
| DLPFC.L-IFG.R | (27,36) | 0.137±0.629 | 0.118±0.515 | 0.173 | 0.863 | 0.951652173913043 |
| DLPFC.L-FPA | (27,37) | -0.019±0.58 | -0.064±0.589 | 0.393 | 0.695 | 0.882866666666667 |
| DLPFC.L-FPA | (27,38) | 0.151±0.62 | 0.085±0.719 | 0.499 | 0.619 | 0.875727272727273 |
| DLPFC.L-IFG.L | (27,39) | -0.072±0.479 | -0.167±0.687 | 0.814 | 0.418 | 0.875727272727273 |
| DLPFC.L-IFG.L | (27,40) | -0.083±0.538 | 0.006±0.727 | -0.702 | 0.484 | 0.875727272727273 |
| DLPFC.L-OFC.R | (27,41) | 0.001±0.635 | -0.001±0.595 | 0.021 | 0.983 | 0.994 |
| DLPFC.L-OFC.R | (27,42) | 0.059±0.658 | -0.126±0.694 | 1.392 | 0.167 | 0.619471698113208 |
| DLPFC.L-OFC.L | (27,44) | 0.074±0.609 | 0.157±0.705 | -0.643 | 0.522 | 0.875727272727273 |
| DLPFC.L-OFC.L | (27,45) | 0.152±0.647 | -0.032±0.577 | 1.52 | 0.132 | 0.619471698113208 |
| FPA-IFG.R | (32,35) | -0.073±0.685 | -0.072±0.722 | -0.008 | 0.994 | 0.994 |
| FPA-IFG.R | (32,36) | 0.066±0.765 | 0.065±0.755 | 0.009 | 0.993 | 0.994 |
| FPA-FPA | (32,37) | 0.076±0.719 | 0.04±0.684 | 0.264 | 0.792 | 0.919268456375839 |
| FPA-FPA | (32,38) | 0.146±0.879 | 0.039±0.75 | 0.666 | 0.507 | 0.875727272727273 |
| FPA-IFG.L | (32,39) | -0.103±0.743 | -0.04±0.633 | -0.462 | 0.645 | 0.875727272727273 |
| FPA-IFG.L | (32,40) | -0.056±0.764 | -0.037±0.712 | -0.131 | 0.896 | 0.951652173913043 |
| FPA-OFC.R | (32,41) | 0.061±0.7 | -0.013±0.756 | 0.518 | 0.605 | 0.875727272727273 |
| FPA-OFC.R | (32,42) | -0.064±0.531 | 0.01±0.734 | -0.58 | 0.563 | 0.875727272727273 |
| FPA-OFC.L | (32,44) | 0.187±0.717 | 0.007±0.654 | 1.332 | 0.186 | 0.619471698113208 |
| FPA-OFC.L | (32,45) | 0.063±0.854 | 0.047±0.738 | 0.1 | 0.92 | 0.965153374233129 |
| IFG.R-IFG.R | (35,36) | -0.023±0.662 | 0.063±0.636 | -0.674 | 0.502 | 0.875727272727273 |
| IFG.R-FPA | (35,37) | -0.173±0.554 | -0.091±0.676 | -0.673 | 0.502 | 0.875727272727273 |
| IFG.R-FPA | (35,38) | 0.042±0.749 | -0.033±0.676 | 0.533 | 0.595 | 0.875727272727273 |
| **IFG.R-IFG.L** | **(35,39)** | **0.055±0.51** | **-0.231±0.662** | **2.452** | **0.016** | 0.264272727272727 |
| IFG.R-IFG.L | (35,40) | -0.173±0.603 | 0.028±0.674 | -1.593 | 0.114 | 0.619471698113208 |
| IFG.R-OFC.R | (35,41) | 0.002±0.653 | -0.028±0.708 | 0.226 | 0.821 | 0.929741721854305 |
| IFG.R-OFC.R | (35,42) | -0.042±0.565 | -0.048±0.632 | 0.055 | 0.957 | 0.985825301204819 |
| IFG.R-OFC.L | (35,44) | 0.116±0.695 | 0.047±0.631 | 0.527 | 0.599 | 0.875727272727273 |
| IFG.R-OFC.L | (35,45) | 0.152±0.643 | -0.041±0.708 | 1.45 | 0.15 | 0.619471698113208 |
| IFG.R-FPA | (36,37) | -0.016±0.615 | 0.07±0.674 | -0.673 | 0.502 | 0.875727272727273 |
| IFG.R-FPA | (36,38) | 0.205±0.734 | 0.14±0.779 | 0.431 | 0.667 | 0.875727272727273 |
| IFG.R-IFG.L | (36,39) | 0.009±0.792 | -0.052±0.685 | 0.419 | 0.676 | 0.875727272727273 |
| IFG.R-IFG.L | (36,40) | 0.086±0.631 | 0.162±0.638 | -0.608 | 0.544 | 0.875727272727273 |
| IFG.R-OFC.R | (36,41) | 0.132±0.668 | 0.215±0.657 | -0.634 | 0.528 | 0.875727272727273 |
| IFG.R-OFC.R | (36,42) | 0.067±0.781 | -0.038±0.671 | 0.734 | 0.465 | 0.875727272727273 |
| IFG.R-OFC.L | (36,44) | 0.231±0.625 | 0.292±0.658 | -0.486 | 0.628 | 0.875727272727273 |
| IFG.R-OFC.L | (36,45) | 0.156±0.558 | 0.068±0.679 | 0.724 | 0.471 | 0.875727272727273 |
| **FPA-FPA** | **(37,38)** | **0.251±0.621** | **-0.066±0.694** | **2.434** | **0.017** | 0.264272727272727 |
| FPA-IFG.L | (37,39) | 0.028±0.678 | -0.159±0.662 | 1.413 | 0.161 | 0.619471698113208 |
| FPA-IFG.L | (37,40) | -0.025±0.711 | -0.193±0.56 | 1.338 | 0.184 | 0.619471698113208 |
| FPA-OFC.R | (37,41) | 0.144±0.756 | -0.131±0.712 | 1.898 | 0.061 | 0.474136363636364 |
| FPA-OFC.R | (37,42) | 0.154±0.603 | -0.039±0.736 | 1.454 | 0.149 | 0.619471698113208 |
| FPA-OFC.L | (37,44) | 0.083±0.808 | 0.071±0.724 | 0.079 | 0.937 | 0.976993902439025 |
| FPA-OFC.L | (37,45) | 0.091±0.721 | -0.133±0.667 | 1.642 | 0.104 | 0.613241379310345 |
| **FPA-IFG.L** | **(38,39)** | **0.09±0.734** | **-0.213±0.633** | **2.251** | **0.027** | 0.3078 |
| FPA-IFG.L | (38,40) | 0.011±0.71 | 0.008±0.648 | 0.026 | 0.979 | 0.994 |
| FPA-OFC.R | (38,41) | 0.22±0.606 | 0±0.681 | 1.737 | 0.085 | 0.565615384615385 |
| FPA-OFC.R | (38,42) | 0.05±0.766 | -0.023±0.687 | 0.51 | 0.611 | 0.875727272727273 |
| FPA-OFC.L | (38,44) | 0.195±0.739 | 0.059±0.734 | 0.939 | 0.35 | 0.83125 |
| FPA-OFC.L | (38,45) | 0.144±0.694 | -0.017±0.792 | 1.096 | 0.276 | 0.733984615384615 |
| IFG.L-IFG.L | (39,40) | -0.1±0.568 | -0.252±0.602 | 1.323 | 0.189 | 0.619471698113208 |
| IFG.L-OFC.R | (39,41) | 0.021±0.584 | -0.159±0.605 | 1.536 | 0.128 | 0.619471698113208 |
| IFG.L-OFC.R | (39,42) | 0.085±0.558 | -0.096±0.765 | 1.365 | 0.175 | 0.619471698113208 |
| IFG.L-OFC.L | (39,44) | 0.079±0.545 | -0.057±0.697 | 1.101 | 0.273 | 0.733984615384615 |
| IFG.L-OFC.L | (39,45) | 0.16±0.628 | -0.105±0.736 | 1.967 | 0.052 | 0.45315 |
| IFG.L-OFC.R | (40,41) | 0.086±0.644 | 0.041±0.729 | 0.33 | 0.742 | 0.912820143884892 |
| IFG.L-OFC.R | (40,42) | 0.099±0.637 | -0.099±0.717 | 1.48 | 0.142 | 0.619471698113208 |
| IFG.L-OFC.L | (40,44) | 0.151±0.561 | 0.055±0.732 | 0.746 | 0.457 | 0.875727272727273 |
| IFG.L-OFC.L | (40,45) | 0.078±0.68 | -0.056±0.738 | 0.957 | 0.341 | 0.826098591549296 |
| OFC.R-OFC.R | (41,42) | 0.117±0.59 | 0.019±0.628 | 0.816 | 0.417 | 0.875727272727273 |
| OFC.R-OFC.L | (41,44) | 0.083±0.531 | 0.114±0.543 | -0.29 | 0.772 | 0.919268456375839 |
| OFC.R-OFC.L | (41,45) | 0.043±0.601 | 0.129±0.658 | -0.692 | 0.491 | 0.875727272727273 |
| OFC.R-OFC.L | (42,44) | 0.108±0.599 | -0.079±0.738 | 1.415 | 0.16 | 0.619471698113208 |
| OFC.R-OFC.L | (42,45) | 0.165±0.721 | 0.039±0.8 | 0.838 | 0.404 | 0.875727272727273 |
| OFC.L-OFC.L | (44,45) | 0.17±0.608 | 0.232±0.718 | -0.475 | 0.636 | 0.875727272727273 |

The values are the means ± standard deviations.

PMC,premotor area;DLPFC,dorsolateral prefrontal cortex;FPA,frontal pole area; IFG,inferior frontal gyrus; OFC,.orbitofrontal cortex; R, right; L, left

**Table S10. Brain–Behavior Correlations**

| **Brain regions** | **Haemodynamic changes** |  | **Δ Accuracy rate** | **Δ Brief-BESTest** |
| --- | --- | --- | --- | --- |
| **DLPFC.L** | **Δ CH22 β value** | r | 0.313 | 0.308 |
|  |  | *P* | **0.034** | **0.038** |
| **IFG.R** | **Δ CH35 β value** | r | 0.297 | 0.04 |
|  |  | *P* | **0.045** | 0.791 |
| **PMC.R-FPA** | **Δ correlation(1,38)** | r | 0.279 | 0.482 |
|  |  | *P* | 0.06 | **0.001** |
| **DLPFC.R-OFC.R** | **Δ correlation(26,41)** | r | 0.382 | -0.056 |
|  |  | *P* | **0.009** | 0.712 |

PMC,premotor area;DLPFC,dorsolateral prefrontal cortex;FPA,frontal pole area; IFG,inferior frontal gyrus; OFC,.orbitofrontal cortex; R, right; L, left；Δ, 6 weeks post-intervention testing minus baseline testing
