# Supplementary material for: Additional Effects of 1‐Hz rTMS Combined With Tai Chi Chuan on Dual‐Task Performance Among Older Adults With Sleep Disorders and MCI: An fNIRS‐Based Randomized Controlled Trial
Source: Brain Behav. 2026 Sep 14;16(9):e71743. doi: 10.1002/brb3.71743 (PMC13573082; doi:10.1002/brb3.71743)
Supplement: Supplementary file 2 [file BRB3-16-e71743-s002.docx]

**Table S1. Coordinates of 45 Channels and 28 Optodes**

|  | **x** | **y** | **z** |
| --- | --- | --- | --- |
| CH01 | 20.655595 | -2.544134 | 10.835575 |
| CH02 | 20.88728 | 0.222812 | 9.711156 |
| CH03 | 20.0995 | 2.987497 | 9.564978 |
| CH04 | 19.585109 | -4.25407 | 10.339864 |
| CH05 | 20.745524 | -1.800553 | 8.297848 |
| CH06 | 20.291206 | 1.232816 | 8.032225 |
| CH07 | 19.324991 | 3.998417 | 8.135273 |
| CH08 | 18.567013 | -5.556118 | 10.04173 |
| CH09 | 19.675038 | -3.510489 | 7.802138 |
| CH10 | 20.14945 | -0.79055 | 6.618917 |
| CH11 | 19.516698 | 2.243735 | 6.602521 |
| CH12 | 18.649134 | 5.060637 | 7.15416 |
| CH13 | 16.993927 | -6.749155 | 9.932754 |
| CH14 | 18.209291 | -5.215183 | 7.74275 |
| CH15 | 19.236736 | -2.844574 | 5.623838 |
| CH16 | 19.240163 | 0.236041 | 5.150416 |
| CH17 | 18.512757 | 3.140314 | 4.974532 |
| CH18 | 17.702798 | 5.8765 | 6.104245 |
| CH19 | 16.636204 | -6.408219 | 7.633773 |
| CH20 | 17.770988 | -4.549268 | 5.56445 |
| CH21 | 18.327449 | -1.817984 | 4.155337 |
| CH22 | 18.236222 | 1.132619 | 3.522427 |
| CH23 | 17.566422 | 3.956177 | 3.924617 |
| CH24 | 14.837257 | -7.349669 | 7.797447 |
| CH25 | 16.260005 | -5.963222 | 5.262304 |
| CH26 | 17.038213 | -3.669368 | 3.36813 |
| CH27 | 17.409888 | -0.640695 | 2.34495 |
| CH28 | 16.921494 | 2.195174 | 2.117793 |
| CH29 | 16.042576 | 5.197731 | 3.387778 |
| CH30 | 14.461058 | -6.904671 | 5.425978 |
| CH31 | 15.52723 | -5.083321 | 3.065984 |
| CH32 | 16.120653 | -2.492079 | 1.557742 |
| CH33 | 16.09516 | 0.421859 | 0.940315 |
| CH34 | 15.397649 | 3.436728 | 1.580953 |
| CH35 | 12.368248 | -7.758388 | 6.567124 |
| CH36 | 13.68508 | -6.443444 | 3.668811 |
| CH37 | 14.431691 | -4.300668 | 1.633052 |
| CH38 | 14.778542 | -1.196678 | 0.453888 |
| CH39 | 14.389577 | 1.853065 | 0.28123 |
| CH40 | 13.403838 | 4.714047 | 1.707644 |
| CH41 | 11.592269 | -7.297161 | 4.809957 |
| CH42 | 12.589541 | -5.660791 | 2.235879 |
| CH43 | 13.08958 | -3.005267 | 0.529198 |
| CH44 | 13.072958 | 0.234528 | -0.205197 |
| CH45 | 12.395766 | 3.130384 | 0.407921 |
| T1 | 21.214748 | -1.25998 | 9.975106 |
| R1 | 20.096441 | -3.828289 | 11.696043 |
| T2 | 19.639187 | 4.26939 | 9.68275 |
| R2 | 20.559813 | 1.705604 | 9.447206 |
| T4 | 19.073776 | -4.679851 | 8.983685 |
| R4 | 18.060249 | -6.432386 | 11.099775 |
| T5 | 20.0226 | 0.760027 | 6.617244 |
| R5 | 20.2763 | -2.341126 | 6.62059 |
| T6 | 18.287472 | 6.39383 | 7.720522 |
| R6 | 19.010796 | 3.727443 | 6.587797 |
| T7 | 15.927604 | -7.065924 | 8.765733 |
| R7 | 17.344805 | -5.750514 | 6.501814 |
| T8 | 18.197172 | -3.348022 | 4.627086 |
| R8 | 18.457726 | -0.287946 | 3.683588 |
| T9 | 18.014719 | 2.553184 | 3.361266 |
| R9 | 17.118124 | 5.359169 | 4.487968 |
| T10 | 15.175205 | -6.175929 | 4.022794 |
| R10 | 13.74691 | -7.633413 | 6.829162 |
| T11 | 16.362051 | -0.993444 | 1.006311 |
| R11 | 15.879254 | -3.990713 | 2.109174 |
| T12 | 14.967028 | 5.036294 | 2.287587 |
| R12 | 15.82827 | 1.837163 | 0.874319 |
| T13 | 10.989585 | -7.883362 | 6.305086 |
| R13 | 12.194954 | -6.710959 | 3.314828 |
| T14 | 12.984128 | -4.610622 | 1.156931 |
| R14 | 13.195032 | -1.399912 | -0.098535 |
| T15 | 12.950884 | 1.868967 | -0.311859 |
| R15 | 11.840649 | 4.3918 | 1.127701 |

CH,channel; T,Transmitter; R,Receiver

**Table S2. Coordinates of 19 ROI channels and their corresponding brain regions.**

| **ROI channels** | **Brain regions** | **x** | **y** | **z** |
| --- | --- | --- | --- | --- |
| CH01 | PMC | 20.655595 | -2.544134 | 10.835575 |
| CH03 | PMC | 20.0995 | 2.987497 | 9.564978 |
| CH04 | PMC | 19.585109 | -4.25407 | 10.339864 |
| CH07 | PMC | 19.324991 | 3.998417 | 8.135273 |
| CH20 | DLPFC | 17.770988 | -4.549268 | 5.56445 |
| CH22 | DLPFC | 18.236222 | 1.132619 | 3.522427 |
| CH26 | DLPFC | 17.038213 | -3.669368 | 3.36813 |
| CH27 | DLPFC | 17.409888 | -0.640695 | 2.34495 |
| CH32 | FPA | 16.120653 | -2.492079 | 1.557742 |
| CH35 | IFG | 12.368248 | -7.758388 | 6.567124 |
| CH36 | IFG | 13.68508 | -6.443444 | 3.668811 |
| CH37 | FPA | 14.431691 | -4.300668 | 1.633052 |
| CH38 | FPA | 14.778542 | -1.196678 | 0.453888 |
| CH39 | IFG | 14.389577 | 1.853065 | 0.28123 |
| CH40 | IFG | 13.403838 | 4.714047 | 1.707644 |
| CH41 | OFC | 11.592269 | -7.297161 | 4.809957 |
| CH42 | OFC | 12.589541 | -5.660791 | 2.235879 |
| CH44 | OFC | 13.072958 | 0.234528 | -0.205197 |
| CH45 | OFC | 12.395766 | 3.130384 | 0.407921 |

PMC,premotor area;DLPFC,dorsolateral prefrontal cortex;FPA,frontal pole area; IFG, inferior frontal gyrus; OFC,.orbitofrontal cortex;ROI channel,interest channel;CH,channel
