# Supplementary material for: Additional Effects of 1‐Hz rTMS Combined With Tai Chi Chuan on Dual‐Task Performance Among Older Adults With Sleep Disorders and MCI: An fNIRS‐Based Randomized Controlled Trial
Source: Brain Behav. 2026 Sep 14;16(9):e71743. doi: 10.1002/brb3.71743 (PMC13573082; doi:10.1002/brb3.71743)
Supplement: Supplementary file 3 [file BRB3-16-e71743-s003.docx]

**Data Sharing Statement**

**Data**

**Additional Information:** Clinical Trials. gov Identifier: ChiCTR2200063274

D**ata available:** Yes

**Data types:** Deidentified participant data

**How to access data:** Contact corresponding author: lzz@fjtcm.edu.cn

**When available:** With publication

**Supporting Documents**

**Document types:** None

**Additional Information**

**Who can access the data:** Researchers whose proposed use of the data has been approved

**Types of analyses:** Academic purpose

**Mechanisms of data availability:** with investigator support
